# Targeting OGG1 and PARG radiosensitises head and neck cancer cells to high-LET protons through complex DNA damage persistence

**DOI:** 10.1038/s41419-024-06541-9

**Published:** 2024-02-17

**Authors:** Maria Rita Fabbrizi, Catherine M. Nickson, Jonathan R. Hughes, Emily A. Robinson, Karthik Vaidya, Carlos P. Rubbi, Andrzej Kacperek, Helen E. Bryant, Thomas Helleday, Jason L. Parsons

**Affiliations:** 1 Institute of Cancer and Genomic Sciences, University of Birmingham, Edgbaston, Birmingham B15 2TT UK; 2 Department of Molecular and Clinical Cancer Medicine, University of Liverpool, Liverpool, L7 8TX UK; 3 Medical School, Edge Hill University, St Helens Road, Ormskirk, L39 4QP UK; 4 Clatterbridge Cancer Centre NHS Foundation Trust, Clatterbridge Road, Bebington, CH63 4JY UK; 5 Sheffield Institute for Nucleic Acids (SInFoNiA), School of Medicine and Population Health, University of Sheffield, Sheffield, S10 2RX UK; 6 Science for Life Laboratory, Department of Oncology and Pathology, Karolinska Institute, Stockholm, Sweden

**Keywords:** Cancer therapy, Cancer

## Abstract

Complex DNA damage (CDD), containing two or more DNA lesions within one or two DNA helical turns, is a signature of ionising radiation (IR) and contributes significantly to the therapeutic effect through cell killing. The levels and complexity of CDD increases with linear energy transfer (LET), however, the specific cellular response to this type of DNA damage and the critical proteins essential for repair of CDD is currently unclear. We performed an siRNA screen of ~240 DNA damage response proteins to identify those specifically involved in controlling cell survival in response to high-LET protons at the Bragg peak, compared to low-LET entrance dose protons which differ in the amount of CDD produced. From this, we subsequently validated that depletion of 8-oxoguanine DNA glycosylase (OGG1) and poly(ADP-ribose) glycohydrolase (PARG) in HeLa and head and neck cancer cells leads to significantly increased cellular radiosensitivity specifically following high-LET protons, whilst no effect was observed after low-LET protons and X-rays. We subsequently confirmed that OGG1 and PARG are both required for efficient CDD repair post-irradiation with high-LET protons. Importantly, these results were also recapitulated using specific inhibitors for OGG1 (TH5487) and PARG (PDD00017273). Our results suggest OGG1 and PARG play a fundamental role in the cellular response to CDD and indicate that targeting these enzymes could represent a promising therapeutic strategy for the treatment of head and neck cancers following high-LET radiation.

## Introduction

Ionising radiation (IR) is used to treat ~50% of all human cancers, where largely conventional X-ray (photon) irradiation is utilised. However, this can create acute and long-term adverse side effects due to the irradiation of normal tissues and organs at risk in proximity to the tumour. In contrast, proton beam therapy (PBT) can more precisely deliver radiation to the tumour due to dose deposition via the Bragg peak [1], limiting the side effect profile. PBT is currently used for the treatment of solid tumours, including head and neck squamous cell carcinoma (HNSCC) [2]. Although, there is significant clinical and biological uncertainty due to increases in linear energy transfer (LET) at and around the Bragg peak, that can lead to changes in biological effectiveness.

The critical cellular target for IR, including photons and PBT, is DNA and the induction of DNA double-strand breaks (DSBs) and complex DNA damage (CDD) are considered the most important in driving the radiobiological response [3, 4]. CDD is defined as two or more DNA lesions within one or two DNA helical turns and represents a significant challenge to the cellular DNA repair machinery, leading to its persistence in cells and tissues several hours post-irradiation [5–7]. Elevated quantities and complexity of CDD are dependent on the LET of the IR, which has been predicted through mathematical modelling [8], although it is technically challenging to measure in biological systems. Experiments using heavy ions have shown DNA DSB repair foci can persist up to 24–48 h post-irradiation and lead to increase in chromosomal aberrations, generating enhanced biological effectiveness [9, 10]. Formation of CDD has similarly been observed in response to PBT through the use of surrogate DNA damage markers [11], and through monitoring DNA damage and repair directly [12, 13]. CDD is unquestionably contributing to the enhanced relative biological effectiveness (RBE) of PBT [14].

Difficulties in detecting and quantifying CDD in cell and tissue models has been an obstacle in determining the cellular DNA damage response (DDR) that is triggered. However, through monitoring cell survival using DNA repair-deficient Chinese Hamster cells, it has been shown that there is a preference for homologous recombination (HR) following high-LET carbon ions compared to protons with a lower LET, although non-homologous end joining (NHEJ) was important for both radiation modalities [15]. Increased preference for HR in response to PBT was observed through enhanced cellular radiosensitivity of RAD51-depleted lung carcinoma cells, associated with delays in resolving of γH2AX foci [16], but also in osteosarcoma cells through persistence in RAD51 and RPA foci [17]. Interestingly, it has been suggested that CDD generated through high-LET Fe ions can inhibit Ku-dependent (classical) NHEJ but not HR or PARP-1-dependent (alternative) NHEJ [18], and supported by other studies suggesting a greater dependence on alternative NHEJ or HR for CDD repair [19, 20]. We have also recently demonstrated that HeLa and HNSCC cells irradiated with PBT at the Bragg peak have a distinct requirement for PARP-1 to maintain cell survival [13], and is required for efficient repair of CDD (measured directly through enzyme-modified comet assays) that is largely DNA single strand break (SSB)-associated. Despite this, further studies are required for identifying key proteins within the cellular DDR that are essential for CDD repair, but which will no doubt depend on the spectrum of the damage through physical (e.g., IR source, dose, and dose rate) and biological (e.g., tumour model and inherent radiosensitivity) factors.

In the current study, through siRNA screening we have identified that 8-Oxoguanine DNA Glycosylase (OGG1) and Poly(ADP-Ribose) Glycohydrolase (PARG) are fundamental for HeLa and HNSCC cell survival post-irradiation with high-LET PBT. We also show that the efficiency of CDD repair is impaired in cells lacking, or containing inhibitors of, OGG1 and PARG. This provides evidence that OGG1 and PARG could represent potential therapeutic targets to enhance the efficacy of high-LET IR in the treatment of HNSCC.

## Materials and methods

### Antibodies, siRNA, and inhibitors

The Human ON-TARGETplus siRNA Library-DNA Damage Response and the siRNA targeting OGG1, PARG, Pol β, and XRCC1, each containing a pool of four different siRNA sequences to help aid knockdown efficiencies, were from Horizon Discovery (Cambridge, UK). The non-targeting control siRNA (AllStars Negative Control siRNA) was from Qiagen (Manchester, UK). The following antibodies were used: γH2AX (05–636; Merck-Millipore, Watford, UK), OGG1, PARG, phosphorylated ATM and phosphorylated DNA-PKcs (ab124741, ab169639, ab81292 and ab18192; Abcam, Cambridge, UK), PARP-1 (sc-53643; Santa Cruz Biotechnology, Heidelberg, Germany), OGG1 for immunofluorescence (NB100-106; Bio-Techne Ltd, Abingdon, UK), Polβ and XRCC1 (kindly provided by G. Dianov) and tubulin (T6199; Sigma-Aldrich, Gillingham, UK). Goat anti-mouse Alexa Fluor 555 or goat anti-rabbit Alexa Fluor 488 secondary antibodies for immunofluorescence were from Life Technologies (Paisley, UK). The OGG1 inhibitor TH5487, as previously described [21], was used at a concentration of 10 µM. The PARG inhibitor PDD00017273, also previously described [22], was used at a concentration of 1 µM.

### Cell culture and irradiation sources

Oropharyngeal squamous cell carcinoma cells (UMSCC6 and UMSCC74A) were kindly provided by Prof T. Carey. Hypopharyngeal squamous cell carcinoma cells (FaDu) were purchased from ATCC (Virginia, USA). All cell lines were routinely cultured as monolayers in 5% CO_2_ at 37 °C as previously described [23], in Dulbecco’s Modified Eagle Medium (DMEM) supplemented with 10% fetal bovine serum, 2 mM L-glutamine, 1× penicillin-streptomycin and 1× non-essential amino acids, except for FaDu cells which were cultured in Modified Eagle Medium (MEM). All cells were authenticated in our laboratory by STR profiling. siRNA knockdowns were performed for 48 h using Lipofectamine RNAiMAX (Life Technologies, Paisley, UK). Irradiation sources are as previously described [12]. In brief, cells were exposed to either low-LET X-rays (100 kV; CellRad X-ray irradiator, Faxitron Bioptics, Tucson, USA), or with a horizontal, passive-scattered proton beam line of 60 MeV maximal energy at the Clatterbridge Cancer Centre. For low-LET proton irradiations, cells were irradiated directly by a ~ 1 keV/µm pristine beam of 58 MeV effective energy (dose rate of ~5 Gy/min). For high-LET proton irradiations, a modulator was utilized to generate a 27 mm spread-out Bragg peak and a 24.4 mm absorber was used to position the cells at the distal edge, corresponding to a mean proton energy of 11 MeV at a dose averaged LET of 12 keV/µm (dose rate of ~5 Gy/min).

### Clonogenic assays

For siRNA screening and following gene depletion for 48 h, HeLa cells were irradiated in 35 mm dishes, harvested and a defined number seeded in triplicate into 6 well-plates. For HNSCC cells, these were plated as single cells into 35 mm dishes ~16 h prior to irradiation. Plating efficiencies for the cells were as followed: HeLa (~40%), FaDu (~20%), UMSCC6 (~15%), and UMSCC74A (~15%). Increasing cell numbers were plated for increasing IR doses to allow for plating efficiencies. Colonies were allowed to grow for 7–10 days, prior to fixing and staining with 6% glutaraldehyde and 0.5% crystal violet for 30 min. Dishes were washed, left to air dry overnight and colonies counted using the GelCount colony analyser (Oxford Optronics, Oxford, UK). Colony counting settings were optimized for both cell lines, based on inclusion of distinct colonies of specific size and intensity, although the same settings were used across the various treatments. Relative colony formation (surviving fraction) was expressed as colonies per treatment level versus colonies that appeared in the untreated control. Results were accumulated from at least three independent biological experiments, apart from the siRNA screen which was from a single experiment (but containing triplicate samples).

### Enzyme-modified neutral comet assay

Detection of DSBs and CDD was achieved using the enzyme-modified neutral comet assay, as recently described [24]. In brief, cells were trypsinised, diluted to 1 × 10^5^ cells/ml, and 250 µl aliquots of the cell suspension were placed into the wells of a 24-well plate which was placed on ice. Cells were irradiated (4 Gy) and embedded on a precoated microscope slide in low-melting agarose (Bio-Rad, Hemel Hempstead, UK). The slides were incubated for up to 4 h at 37 °C in a humidified chamber to allow for DNA repair, prior to cell lysis in buffer containing 2.5 M NaCl, 100 mM EDTA, 10 mM Tris-HCl pH 10.5, 1% N-lauroylsarcosine, 1% DMSO and 1% (v/v) Triton X-100. Slides were washed three times with enzyme reaction buffer (40 mM HEPES-KOH, 100 mM KCl, 0.5 mM EDTA and 0.2 mg/ml BSA, pH 8.0), and then incubated with either buffer alone (mock treated; revealing levels of DNA DSBs) or with buffer containing 5 pmol OGG1, 6 pmol NTH1 and 0.6 pmol APE1 (enzyme treated; revealing levels of DNA DSBs plus CDD) for 1 h at 37 °C in a humidified chamber. Following treatment, slides were placed in cold electrophoresis buffer (1 × TBE buffer (pH 8.3)) in the dark for 25 min to allow the DNA to unwind, prior to electrophoresis at 25 V, ~20 mA for 25 min. Slides were washed three times with 1 × PBS before allowing to dry overnight. Slides were rehydrated for 30 min in water (pH 8.0), stained for 30 min with SYBR Gold (Life Technologies, Paisley, UK) diluted 1:10,000 in water (pH 8.0) and again dried overnight. Cells (50 per slide, in duplicate) were analysed from the dried slides using the Komet 6.0 image analysis software (Andor Technology, Belfast, Northern Ireland) and % tail DNA values averaged from at least three independent biological experiments.

### Immunofluorescence

Measurement of DNA repair protein foci (γH2AX and OGG1) were examined as previously described [25]. In brief, cells were grown on 13 mm circular coverslips until ~70–80% confluent, irradiated at 4 Gy and incubated for up to 24 h in 5% CO_2_ at 37 °C to allow for DNA repair. Cells were washed with PBS at room temperature for 5 min, before being fixed using 10% formalin for 10 min. Cells were permeabilised with 0.2% Triton X-100 in PBS for 10 min, washed three times with 0.1% Tween-20 in PBS for 10 min, and blocked to avoid non-specific staining via incubation with 2% BSA in PBS for 30 min at room temperature on a rocking platform. γH2AX and OGG1 antibodies in 2% BSA were subsequently added and coverslips incubated overnight at 4 °C. Following three washes with PBS, coverslips were incubated with either goat anti-mouse Alexa Fluor 555 or goat anti-rabbit Alexa Fluor 488 secondary antibodies in 2% BSA for 1 h at room temperature in the dark. Finally, samples were washed with PBS for 10 min on a rocking platform and mounted on a microscope slide using Fluoroshield containing DAPI (Sigma-Aldrich, Gillingham, UK). Cells were examined using an Olympus BX61 fluorescent microscope with a Photometrics CoolSNAP HQ2 CCD camera. MicroManager software was used to capture images (~20 images/cell line/antibody).

### Immunoblotting

Whole cell extracts were prepared, separated by SDS-PAGE electrophoresis and analysed by quantitative immunoblotting using the Odyssey image analysis system (Li-cor Biosciences, Cambridge, UK), as previously described [25].

### Statistical Analysis

All experiments were performed in at least triplicate as separate independent, biological experiments. Statistical analysis of clonogenic survival data was performed using the CFAssay for R package [26], which uses the linear-quadratic model (LQ model) to compare different treatment responses across increasing radiation doses. Statistical analysis of DNA damage quantified through neutral comet assays and γH2AX/OGG1 foci through immunofluorescence staining was performed using either a one-sample or two-sample *t* test.

## Results

### Screening for specific DDR proteins involved in response to high-LET protons

**Fig. 1:**
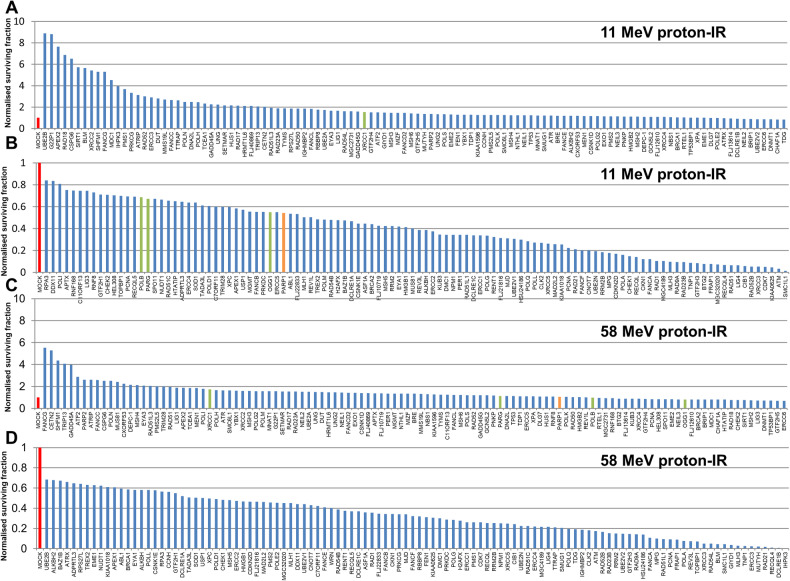
Screening of DDR enzymes involved in cell survival following low- and high-LET protons. HeLa cells were treated with a pool of four siRNA oligonucleotides targeting individual DDR genes for 48 h, and then irradiated with either (**A**, **B**) 2 Gy high-LET protons or (**C**, **D**) 2 Gy low-LET protons. Clonogenic survival of cells was analysed from a single experiment (using triplicate samples) and normalised against the mock treated control (red bar) which was set to 1.0 (equivalent to ~40% cell survival post-irradiation). Surviving fraction of cells following PARP-1 (orange bar), OGG1, PARG, Pol β, and XRCC1 (all green bars) are highlighted.

We performed an siRNA screen of ~240 DDR proteins analysing the survival of HeLa cells after a single dose of PBT, with a particular focus on relatively high-LET protons that generates increased levels and complexity of CDD [12, 13]. Survival was normalized to the irradiated mock-treated sample (generating ~40% cell survival, red bar) which was set to 1.0. The data demonstrates a variability in cell survival in the absence of each DDR protein following relatively high-LET protons (12 keV/µm; Fig. 1A, B), versus low-LET protons (1 keV/µm; Fig. 1C, D, Supplementary Fig. 1). We initially identified candidates whose depletion reduced cell survival by >30% following high-LET protons compared to the mock-treated control, but which comparatively had no significant impact in radiosensitising cells in response to low-LET protons (Supplementary Table 1). From this independent screen, we confirmed that depletion of PARP-1 (Fig. 1B, orange bar) radiosensitised cells to high-LET protons but not to low-LET protons (Fig. 1C), which we demonstrated previously [13]. We also found that depletion of PARG, which degrades poly(ADP-ribose) chains generated by PARP proteins, as well as the base excision repair (BER) enzymes OGG1 and DNA polymerase β (Pol β, green bars) can reduce cell survival by 32–45% compared the mock-treated control specifically following high-LET protons. Interestingly, no apparent effect on survival post-PBT was observed following depletion of the BER scaffold protein XRCC1 (Fig. 1A, C). It was noticeable that certain enzymes involved in DSB repair, such as ATM, CHK1, DNA polymerase θ, DNA-PKcs, and DNA ligase IV when depleted caused radiosensitisation to both high and low-LET PBT.

### Identification and validation of selected DDR enzymes in modulating cell survival in response to high-LET IR

**Fig. 2:**
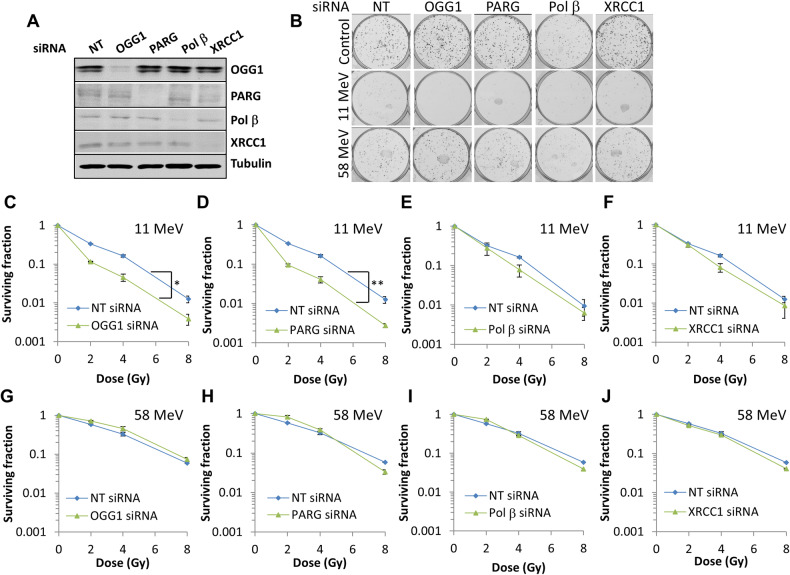
Knockdown of OGG1 or PARG leads to increased radiosensitivity of HeLa cells in response to high-LET protons. HeLa cells were treated with a pool of four siRNAs targeting OGG1, PARG, Pol β, XRCC1 or a non-targeting (NT) control siRNA for 48 h. **A** Whole cell extracts were prepared and analysed by immunoblotting with the indicated antibodies. **B**–**J** Following gene knockdowns, cells were irradiated with increasing doses of (**C**–**F**) high-LET protons or (**G**–**J**) low-LET protons, and clonogenic survival of cells was analysed from three independent experiments. Shown is the mean surviving fraction ± S.E. **B** Respective images of colonies formed from unirradiated (Control) cells, and those following a 4 Gy dose (double the numbers of cells seeded) of relatively high and low-LET protons under the various siRNA knockdowns are shown. *p < 0.002, **p < 0.0001 as analysed by the CFAssay.

Following siRNA screening, we focussed on validating whether depletion of selected enzymes (OGG1, PARG, Pol β, but also XRCC1) can cause a decrease in cell survival following high-LET protons, whilst having no impact in response to low-LET protons, in both HeLa and HNSCC cells. We firstly confirmed the efficiency of the siRNA in supressing levels of the appropriate proteins in HeLa cells (Fig. 2A). We were then able to confirm that OGG1 and PARG siRNA led to significantly reduced cell survival compared to the non-targeting (NT) control siRNA-treated cells in response to high-LET protons (Fig. 2B–D; *p* < 0.002 and *p* < 0.0001 for OGG1 and PARG siRNA, respectively), but not to low-LET protons (Fig. 2B, G, H). Depletion of XRCC1 or Pol β versus the NT control had a much milder effect on HeLa cell survival after high-LET radiation (Fig. 2E and F; *p* < 0.03 for Pol β siRNA, non-significant for XRCC1 siRNA), and did not alter the cellular response to low-LET protons (Fig. 2I, J). We also analysed depletion of OGG1, PARG, Pol β and XRCC1 on survival of HeLa cells following X-rays (Supplementary Fig. 2A–D), but where no significant radiosensitisation was observed.

**Fig. 3:**
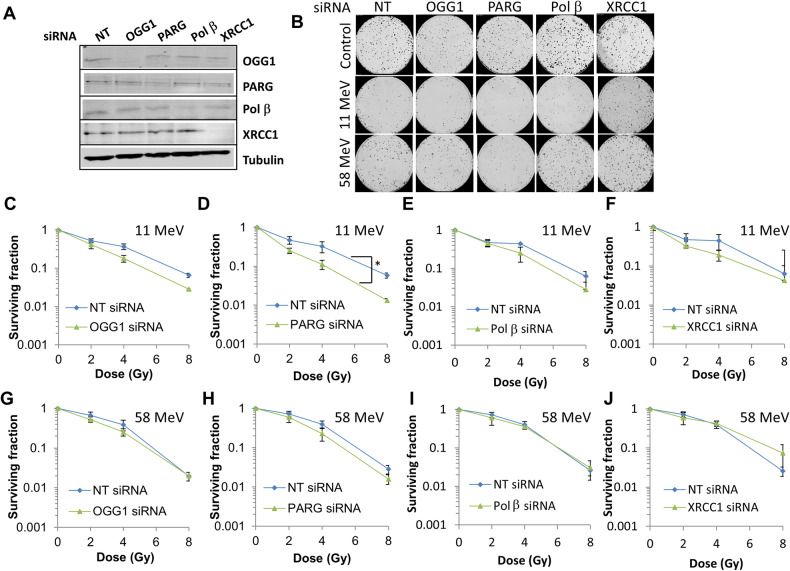
Knockdown of OGG1 or PARG leads to increased radiosensitivity of FaDu cells in response to high-LET protons. FaDu cells were treated with a pool of four siRNAs targeting OGG1, PARG, Pol β, XRCC1, or a non-targeting (NT) control siRNA for 48 h. **A** Whole cell extracts were prepared and analysed by immunoblotting with the indicated antibodies. **B**–**J** Following gene knockdowns, cells were irradiated with increasing doses of (**C**–**F**) high-LET protons or (**G**–**J**) low-LET protons, and clonogenic survival of cells was analysed from three independent experiments. Shown is the mean surviving fraction ± S.E. **B** Respective images of colonies formed from unirradiated (Control) cells, and those following a 4 Gy dose (double the numbers of cells seeded) of relatively high and low-LET protons under the various siRNA knockdowns are shown. *p < 0.0004 as analysed by the CFAssay.

**Fig. 4:**
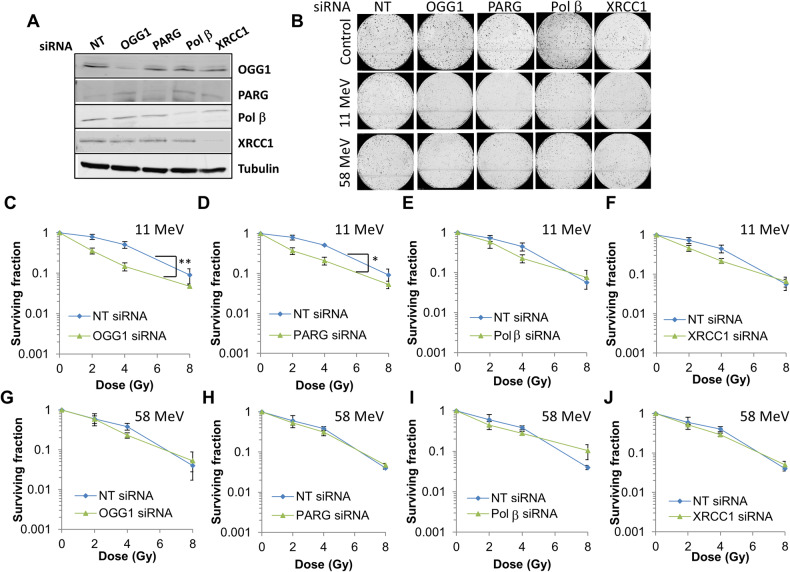
Knockdown of OGG1 or PARG leads to increased radiosensitivity of UMSCC6 cells in response to high-LET protons. UMSCC6 cells were treated with a pool of four siRNAs targeting OGG1, PARG, Pol β, XRCC1, or a non-targeting (NT) control siRNA for 48 h. (**A** Whole cell extracts were prepared and analysed by immunoblotting with the indicated antibodies. **B**–**J** Following gene knockdowns, cells were irradiated with increasing doses of (**C**–**F**) high-LET protons or (**G**–**J**) low-LET protons, and clonogenic survival of cells was analysed from three independent experiments. Shown is the mean surviving fraction ± S.E. **B** Respective images of colonies formed from unirradiated (Control) cells, and those following a 4 Gy dose (double the numbers of cells seeded) of relatively high and low-LET protons under the various siRNA knockdowns are shown. **p < 0.003, *p < 0.0004 as analysed by the CFAssay.

In HNSCC cells, utilising FaDu and UMSCC6 (originating from the hypopharynx and oropharynx, respectively), we confirmed again the effectiveness of the same siRNA sequences in supressing protein levels of OGG1, PARG, Pol β and XRCC1 (Figs. 3A and 4A, respectively). Similar to HeLa cells, OGG1 and PARG siRNA knockdown was found to significantly reduced survival of FaDu (Fig. 3B–D; *p* < 0.08 and *p* < 0.0004 for OGG1 and PARG siRNA, respectively) and UMSCC6 (Fig. 4B–D; *p* < 0.0004 and *p* < 0.003 for OGG1 and PARG siRNA, respectively) compared to NT control siRNA-treated cells specifically in response to high-LET radiation. Radiosensitisation of UMSCC6 cells was still apparent despite the cells harbouring wild-type p53, whereas p53 levels are very low in HeLa and mutated in FaDu. In contrast, an absence of OGG1 and PARG had no significant impact on the response of FaDu (Fig. 3G, H) or UMSCC6 (Fig. 4G, H) to low-LET protons. XRCC1 or Pol β siRNA had a much milder impact than OGG1 or PARG depletion on survival of FaDu (Fig. 3E, F) and UMSCC6 (Fig. 4E, F) to high-LET protons, and which was not significant compared to NT control siRNA-treated cells. XRCC1 or Pol β depletion additionally had no significant impact on survival of FaDu (Fig. 3I, J) and UMSCC6 (Fig. 4I, J) following low-LET protons. Furthermore, depletion of OGG1, PARG, Pol β, or XRCC1 had no impact on the radiosensitivity of HNSCC cells, specifically UMSCC74A (Supplementary Fig. 2E–H), UMSCC6 (Supplementary Fig. 2I–L) and FaDu (Supplementary Fig. 2M–P), to X-rays. Cumulatively, these data confirmed that OGG1 and PARG are required for maintaining survival of HeLa and HNSCC cells in response to relatively high-LET protons.

### OGG1 and PARG depletion cause CDD to persist after high-LET protons

**Fig. 5:**
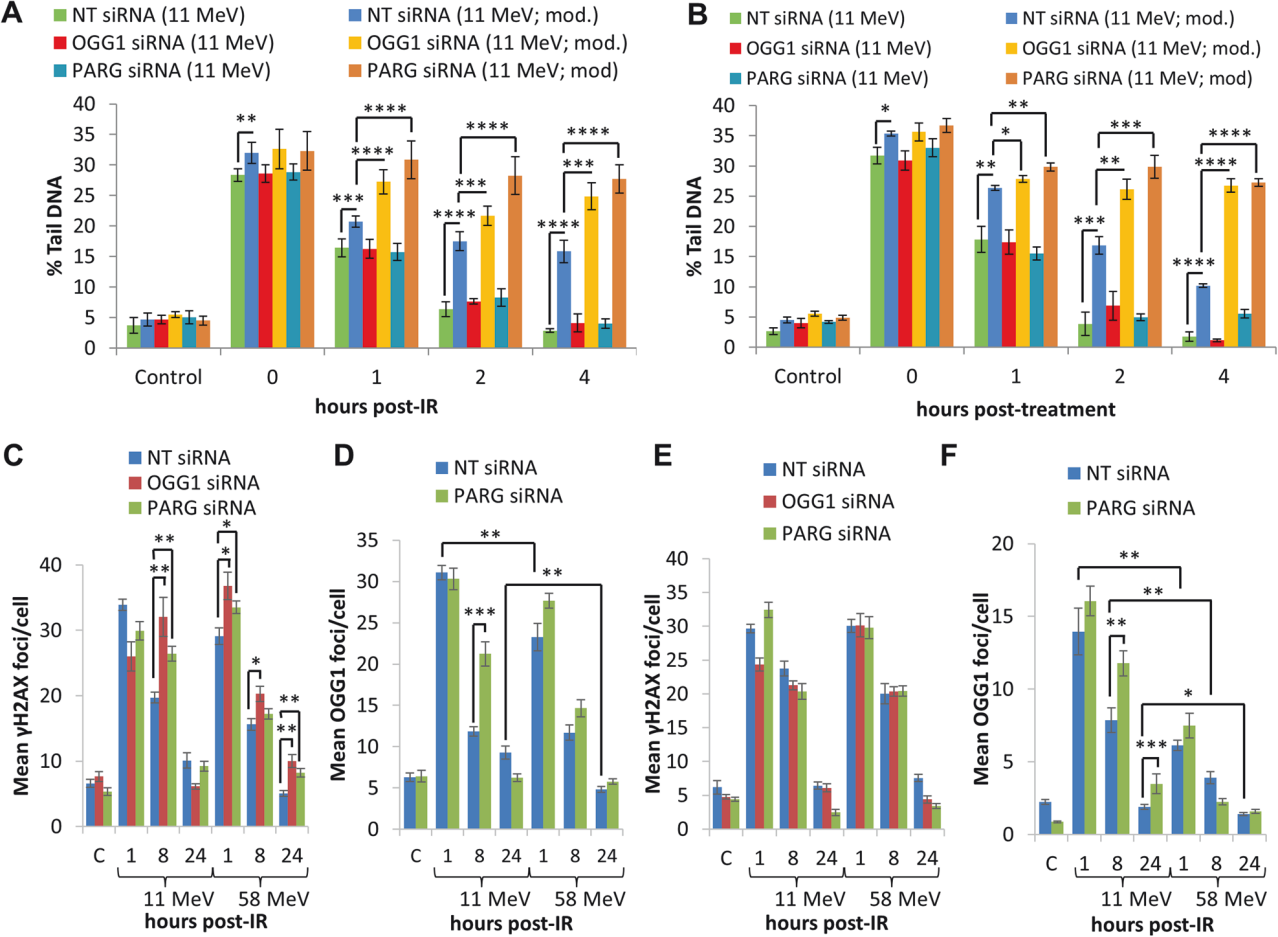
OGG1 and PARG are required for efficient repair of CDD induced by high-LET protons. **A** HeLa or (**B**) FaDu cells were treated with siRNA targeting OGG1 or PARG along with a non-targeting (NT) control siRNA for 48 h. Cells were irradiated with 4 Gy high-LET protons and DNA damage measured at various time points post-IR by the enzyme modified neutral comet assay following incubation in the absence (revealing DSBs) or presence (revealing CDD; as indicated by mod) of the recombinant enzymes APE1, NTH1 and OGG1. Shown is the mean % tail DNA ± S.D. *p < 0.05, **p < 0.01, ***p < 0.001, ****p < 0.0001 as analysed by a one sample *t*-test. Alternatively, cells were irradiated with 4 Gy low- or high-LET protons and (**C**, **E**) γH2AX or (**D**, **F**) OGG1 foci analysed by immunostaining. Shown is the mean γH2AX/OGG1 foci per nuclei ±S.D. *p < 0.01, **p < 0.005, ***p < 0.0005 as analysed by a one sample *t*-test.

We previously demonstrated that relatively high-LET protons lead to increased levels and persistence of CDD that contributes to the enhanced RBE compared to low-LET protons, and that PARP-1 is essential for CDD repair [12, 13]. Using the enzyme-modified neutral comet assay, but in the absence of enzyme-modification where DSB levels are assessed, neither OGG1 or PARG depletion in HeLa cells had any impact on the levels and kinetics of DSB repair induced by high-LET protons compared to NT control siRNA treated cells (Fig. 5A; compare green and red or turquoise bars). In keeping with our previous findings, enzyme modification revealed that CDD formation is evident immediately following high-LET protons (Fig. 5A; compare green and blue bars at time 0). In addition, we now show that CDD significantly persists in HeLa cells lacking either OGG1 or PARG at 1–4 h post-irradiation in comparison to NT control siRNA treated cells (Fig.5A; compare blue and yellow or brown bars). These data were replicated in HNSCC cells, specifically FaDu and UMSCC74A cells (Fig. 5B and Supplementary Fig. 3). Collectively, this demonstrates the importance of both OGG1 and PARG in promoting efficient repair of high-LET proton-induced CDD.

To further support our data, we analysed γH2AX foci as a surrogate marker of DSBs. We observed some significant differences in levels of γH2AX foci at 8 h after irradiation with high-LET protons in cells depleted of either OGG1 or PARG compared to NT siRNA control-treated HeLa (Fig. 5C), although similar elevations at 1–24 h post-irradiation were also seen in response to low-LET protons. In contrast in FaDu cells (Fig. 5E and Supplementary Fig. 4), there were no statistically significant changes in γH2AX foci in cells depleted of OGG1 or PARG following both high or low-LET protons, highlighting that repair of DSBs is largely unaffected in these cells. Using OGG1 foci as a potential surrogate marker of CDD [27], we observed significantly higher levels of OGG1 foci at 1 and 24 h post-irradiation in HeLa (Fig. 5D), and at 1–24 h post-irradiation in FaDu (Fig. 5F and Supplementary Fig. 4) cells treated with high versus low-LET protons. These data are consistent with OGG1 being present at DNA damage sites after high-LET protons which take a longer time to resolve and are therefore complex in nature. Using PARG siRNA, we discovered this caused a further statistically significant increase in OGG1 foci at 8 h post-irradiation in HeLa (Fig. 5D) and at both 8 and 24 h post-irradiation in FaDu (Fig. 5F and Supplementary Fig. 4), following high-LET protons compared to NT siRNA treated cells. This indicates that OGG1 persists at high-LET proton-induced CDD sites, and that PARG is required for enhancing CDD repair efficiency.

### Inhibition of OGG1 and PARG also leads to CDD persistence after high-LET protons

**Fig. 6:**
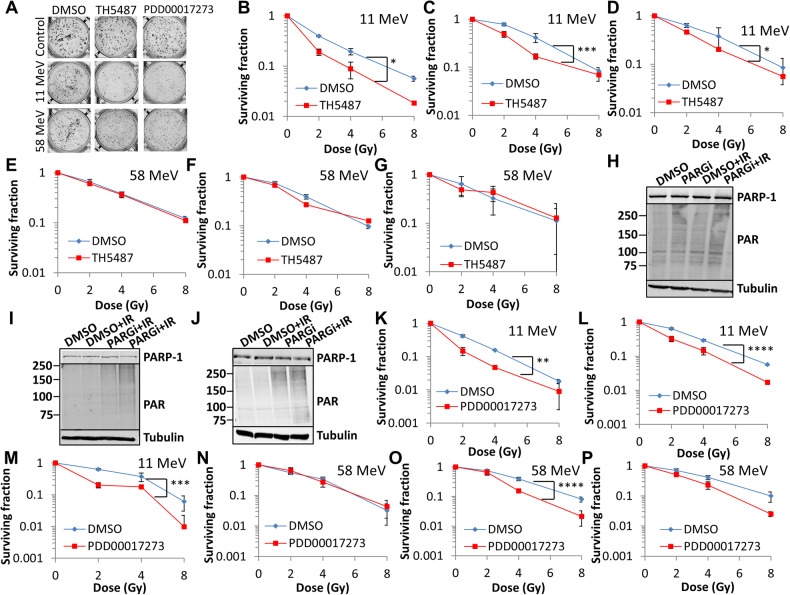
Inhibition of OGG1 or PARG leads to increased radiosensitivity of cells to high-LET protons. **A**, **B**, **E** HeLa, **C**, **F** FaDu or (**D**, **G**) UMSCC6 cells were treated with 10 µM TH5487 or DMSO for 16 h. Cells were then irradiated with increasing doses of (**B**–**D**) high-LET protons or (**E**–**G**) low-LET protons, and clonogenic survival of cells was analysed from three independent experiments. Shown is the mean surviving fraction ± S.E. **B** Respective images of colonies formed from HeLa cells at a 4 Gy dose (double the numbers of cells seeded) of relatively high and low-LET protons under the various drug treatments are shown. **H**, **K**, **N** HeLa, (**I**, **L**, **O**) FaDu, or (**J**, **M**, **P**) UMSCC6 cells were treated with 1 µM PDD00017273 (PARGi) or DMSO for 16 h. **H**–**J** Cells were unirradiated or irradiated (4 Gy X-rays for 15 min), whole cell extracts were prepared and analysed by immunoblotting with antibodies raised against PARP-1, poly(ADP-ribose) (PAR) or tubulin as a loading control. Cells were also irradiated with increasing doses of (**K**–**M**) high-LET protons or (**N**–**P**) low-LET protons, and clonogenic survival of cells was analysed from three independent experiments. Shown is the mean surviving fraction±S.E. *p < 0.04, **p < 0.01, ***p < 0.002, ****p < 0.0001 as analysed by a one sample *t-*test.

We next examined specific inhibitors for OGG1 (TH5487; [21]) and PARG (PDD00017273, [22]). TH5487 caused a marked reduction in survival of HeLa (Fig. 6A, B; *p* < 0.04), FaDu (Fig. 6C; *p* < 0.0007) and UMSCC6 (Fig. 6E; *p* < 0.04) cells compared to DMSO control-treated cells after high-LET protons. In contrast, no significant impact of OGG1 inhibition on the survival of HeLa (Fig. 6E), FaDu (Fig. 6F), and UMSCC6 (Fig. 6G) cells following low-LET protons was observed. Similarly, no sensitisation of HeLa and HNSCC cells was observed in the presence of TH5487 with X-rays (Supplementary Fig. 5A, C, E, G). Utilising PDD00017273, we proved that this is effective in supressing PARG activity in HeLa, FaDu, and UMSCC6 (Fig. 6H-J, respectively) cells, leading to increase poly(ADP-ribosyl)ation. PARG inhibition in combination with high-LET protons was observed to significantly reduce the survival of HeLa (Fig. 6K; *p* < 0.01), FaDu (Fig. 6L; *p* < 0.0001) and UMSCC6 (Fig. 6M; *p* < 0.002) cells compared to the DMSO control. No enhancement of radiosensitivity of HeLa cells with PDD00017273 was observed after low-LET protons (Fig. 6N), or in HeLa and HNSCC cells after X-rays (Supplementary Fig. 5B, D, F, H). Interestingly, inhibition of PARG was found to radiosensitise FaDu (Fig. 6O; *p* < 0.0001) and UMSCC6 cells (Fig. 6P; *p* = 0.12) to low-LET protons, suggests that PARG inhibition in HNSCC cells may have more of a general radiosensitisation role.

**Fig. 7:**
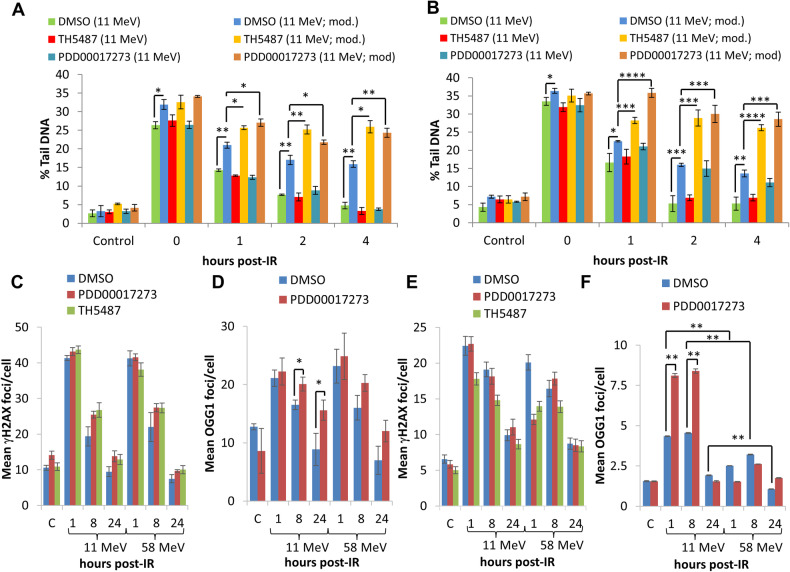
Inhibition of OGG1 and PARG leads to delays in repair of CDD induced by high-LET protons. **A** HeLa or (**B**) FaDu cells were treated with 10 µM TH5487 or 1 µM PDD00017273 along with DMSO as a control for 16 h. Cells were irradiated with 4 Gy high-LET protons and DNA damage measured at various time points post-IR by the enzyme modified neutral comet assay following incubation in the absence (revealing DSBs) or presence (revealing CDD; as indicated by mod) of the recombinant enzymes APE1, NTH1 and OGG1. Shown is the mean % tail DNA ± S.D. *p < 0.05, **p < 0.01, ***p < 0.001, ****p < 0.0001 as analysed by a one sample *t*-test. Alternatively, cells were irradiated with 4 Gy low- or high-LET protons and (**C**, **E**) γH2AX or (**D**, **F**) OGG1 foci analysed by immunostaining. Shown is the mean γH2AX/OGG1 foci per nuclei ±S.D. *p < 0.05, **p < 0.0001 as analysed by a one sample *t*-test.

Using the enzyme-modified comet assay, TH5487 or PDD00017273, compared to the DMSO treated control, had no effect on the levels and repair of DSBs induced by high-LET protons in HeLa cells, as revealed in the absence of enzyme modification (Fig. 7A; compare green, red and turquoise bars). Similar observations were seen in FaDu cells (Fig. 7B; compare green and red bars), although PARG inhibition did cause a modest increase in DSB levels at 2–4 h post-irradiation (Fig. 7B; compare green and turquoise bars). In contrast, high-LET proton-induced CDD levels were significant higher and more persistent at 1–4 h post-treatment in the presence of either TH5487 (Fig. 7A; compare blue and yellow bars) or PDD00017273 (Fig. 7A; compare blue and brown bars) relative to the DMSO control in HeLa, FaDu (Fig. 7B; compare blue and yellow or brown bars), as well as in UMSCC74A cells (Supplementary Fig. 6; compare blue and yellow or brown bars). These data are strengthened by analysis of DNA repair foci, which revealed that levels and kinetics of γH2AX foci as a marker of DSBs were largely unaffected by PDD00017273 or TH5487, compared to the DMSO control, following high-LET protons in HeLa (Fig. 7C) and FaDu (Fig. 7E and Supplementary Fig. 7) cells. OGG1 and PARG inhibition also had no impact on γH2AX foci in response to low-LET protons in these cells. Similarly, there was no apparent difference in the phosphorylation-dependent activation of the major kinases, ATM and DNA-PKcs, in HeLa and FaDu cells following OGG1 and PARG inhibition in response to either low- or high-LET protons (Supplementary Fig. 8A, B). There was also no strong evidence to suggest that apoptosis was significantly induced, measured through monitoring of full length and cleaved PARP-1 protein, in cells treated with the inhibitors following proton irradiation. In contrast, PDD00017273 led to an increase and persistence of OGG1 foci (as a surrogate CDD marker) after treatment with high-LET protons compared to DMSO control-treated cells, which was statistically significant at 8 and 24 h post-irradiation in HeLa (Figs. 7D) and 1 and 8 h post-irradiation in FaDu (Fig. 7F and Supplementary Fig. 7). No effects of PARG inhibition on OGG1 foci were seen in response to low-LET protons in both cells. We also analysed the effects of OGG1 and PARG inhibition on cell cycle checkpoint activation. We found no evidence for a significant difference in the activation of the G2/M checkpoint in FaDu cells following low versus high-LET radiation (Supplementary Fig. 9A, B). Furthermore, whilst OGG1 inhibition through TH5487 caused an apparent decrease in the accumulation of G2/M cells post-irradiation, this was not significantly different compared to DMSO-treated cells and there were no apparent differences observed in the responses between low- and high-LET protons. Similarly, PARG inhibition through PDD00017273 had no impact on cell cycle checkpoint activation comparing low- and high-LET protons, suggesting that this biological end-point is not directly responsible for the enhanced radiosensitivity of PARG and OGG1-inhibited cells to high-LET protons. Collectively, this evidence supports an important role for OGG1 and PARG in the efficient repair of CDD.

## Discussion

CDD is known to be a significant contributor to IR-induced cell killing, due to the inability of cells to efficiently repair the damage [4, 5, 7]. Formation of CDD is particularly important to higher LET IR, and where evidence that PBT can lead to increases in CDD through dose deposition at and around the Bragg peak [1]. Indeed, we have recently demonstrated that relatively high-LET protons generate CDD that persists for several hours post-irradiation, and which enhances the radiobiological effect [12]. Additionally, we have shown that proteins including USP6, PARP-1, and USP9X play significant roles in maintaining cell survival following high-LET protons mediated through promoting CDD repair or by controlling centrosome stability [13, 28]. We now demonstrate that siRNA depletion or specific inhibitors of OGG1 and PARG also sensitise HeLa and selected HNSCC cells specifically in response to high-LET protons, and which similar to PARP-1, is a phenotype related to supression of CDD repair.

We have previously shown in HeLa and HNSCC cells that the levels and repair of DSB generated after high- versus low-LET protons does not differ, but that there are differences in SSB repair following relatively high-LET proton irradiation suggesting that CDD is largely SSB associated [12]. We then identified PARP-1 as playing a key role in CDD repair and maintaining cell survival under these conditions [13], and which is supported by our current study revealing that PARG depletion/inhibition plays a similar role, suggesting that poly(ADP-ribosyl)ation controlled by PARP enzymes and PARG are critical in this process. Interestingly, PDD00017273 has previously been shown to radiosensitise breast epithelial adenocarcinoma cells, albeit following low-LET radiation [29]. We also observed radiosensitisation of HNSCC (FaDu and UMSCC6) cells with low-LET protons in the presence of PDD00017273, although not in response to X-rays. PARP-1 has a well-established role in BER and SSB repair where the generation of poly(ADP-ribose) polymers acts as a binding platform for recruitment of downstream DNA repair proteins that contain PAR binding motifs [30]. Given the complex nature of CDD induced by high-LET protons, which can consist of different combinations of oxidative DNA base damage, DSBs and SSBs, this suggests that poly(ADP-ribosyl)ation is essential for the recruitment and/or retention of DNA repair proteins at these sites to efficiently coordinate CDD repair. It is also possible that this mechanism is required for chromatin remodelling stimulated through poly(ADP-ribosyl)ation of histones. Nevertheless, the lack of observed impact of high-LET protons on DSB repair efficiency suggests an increase in the amount of non-DSB associated CDD dependent on PARP-1/PARG. Interestingly, mathematical modelling has predicted that the levels of complex SSB are relatively static or decrease with increasing LET, which is more evident for high-LET α-particles [8, 31]. It is important to point out that experiments from this current study are acquired using 1 keV/µm and 12 keV/µm (low versus relatively high-LET, respectively), but also that the simulations may not accurately reflect the spectrum of CDD in the more complex environment within cells. Despite this, it is acknowledged that non-DSB CDD is challenging to repair by the BER pathway [5], and could be converted to more toxic DSBs during lesion processing, as shown through overexpression of OGG1 (but also NTH1) [32]. Our utilisation of OGG1, along with NTH1 and APE1, in the enzyme-modified neutral comet assay to reveal CDD sites suggests that oxidative DNA base damage and SSBs are the major components of these non-DSB CDD sites. However, more detailed investigation is required to fully understand the precise nature of CDD, but also to explore the reliance on PARG and PARP-1 for their relatively efficient repair that maintains cellular resistance to high-LET protons.

Similar to PARP-1, PARG is considered to play a pivotal role in resolving stalled replication forks to prevent formation of toxic DSBs [33, 34], raising the possibility that lack of reversal of PARP activation at SSBs or stalled forks is responsible for radiosensitisation to high-LET induced CDD. These precise mechanistic details require further investigation. In tumour cells lacking HR proficiency, PARG inhibition appears to cause synthetic lethality [22, 35–37], in a similar vein to the archetypal synthetic lethality of PARP inhibitors in BRCA1/2-deficient breast cancer cells [38, 39]. Our recent data has also shown that the PARP inhibitors olaparib and talazoparib can synergistically act with low-LET photons and protons to radiosensitise HNSCC cells grown as 3D spheroids, and where greater radiosensitisation occurred in HR-deficient compared to HR-proficient cells [40]. Therefore, two key factors involved in the effectiveness of PARP/PARG inhibition in tumour cell radiosensitisation appear to be HR proficiency, but also the LET (and CDD-dependence) of the radiation. Nevertheless, our data acquired in HR-proficient HeLa cells would suggest a lethal partnership between CDD induced by high-LET protons, and PARG (or PARP) inhibition, that lead to further CDD persistence and likely increased chromosomal aberrations that trigger cell death.

OGG1 is the major DNA glycosylase enzyme responsible for the excision of 8-oxoguanine lesions during BER. Our observation that OGG1 depletion/inhibition leads to enhanced radiosensitivity of HeLa and HNSCC cells to relatively high-LET protons suggests that 8-oxoguanine residues are a major component of CDD sites, along with directly induced SSBs, and that OGG1 appears essential for their repair. This aligns with our utilisation of recombinant OGG1 protein to reveal CDD in the enzyme-modified neutral comet assay, but also that persistent OGG1 foci are present in HeLa and FaDu cells irradiated with high-LET protons. Co-localisation of OGG1 with γH2AX has previously been examined following radiation of increasing LET, to demonstrate elevated levels of complex DSBs [27]. Whilst we see no evidence of a significant difference in levels or repair of DSBs through γH2AX foci and comet analysis comparing relatively high versus low-LET protons, possible co-localisation of OGG1 at DSB sites under our experimental conditions is required. Interestingly, it has very recently been described that BRCA1-deficient breast cancer cells are sensitised to TH5487 demonstrating a new synthetic lethal partnership between HR deficiency and OGG1 inhibition [41]. The relative contribution that HR plays in the response to high-LET radiation, and the interplay with OGG1-dependent BER, also requires further investigation. Intriguingly, there is also evidence suggesting that OGG1 binds directly to PARP-1 to stimulate its poly(ADP-ribosyl)ation activity [42], which would likely be further controlled by PARG. This would suggest a close relationship between PARP-1, PARG and OGG1 in the repair of oxidative DNA damage, but also in the repair of CDD induced by high-LET protons.

The involvement of DSB and other DDR proteins following high-LET protons still needs further investigation. From our DDR screen, an siRNA knockdown of ATM, CHK1, DNA polymerase θ, DNA-PKcs and DNA ligase IV were shown to radiosensitise HeLa cells to both low and high-LET protons and therefore were not investigated further as being LET and therefore CDD specific. It has been heavily debated that there is an increased reliance on HR for repair of DSBs in response to higher LET radiation [7] through experiments with cells irradiated with Bragg peak protons [16, 43, 44], and similar data has been observed with high-LET iron ions [45]. In contrast, it was shown that NHEJ-deficient cells were the most radiosensitive to both proton and higher LET carbon ions compared to wild type and HR-deficient cells [15], and persistence of γH2AX foci which were larger in size in NHEJ-deficient cells following protons has been observed [46]. Despite this, direct measurement of CDD was not conducted, and it is nevertheless difficult to compare studies performed using different cell models, radiation types and energies/LET. Therefore, a more systematic and comparable approach needs to be taken.

In conclusion, we provide evidence that OGG1 and PARG, in addition to our previous identification of PARP-1, play a critical role in IR-induced CDD repair. We also demonstrate that OGG1 and PARG inhibitors can exacerbate the impact of high-LET protons in tumour cell killing, and therefore could represent a novel therapeutic strategy, such as in the treatment of HNSCC. However, further research particularly using more advanced preclinical models, such as patient-derived organoids and xenografts in mice, are necessary.

### Supplementary information

Supplementary Data

Original Data File

## Data Availability

Source data are provided within this paper. Any other data will be made available from the corresponding author upon reasonable request.
