# Supplementary material for: Targeting OGG1 and PARG radiosensitises head and neck cancer cells to high-LET protons through complex DNA damage persistence
Source: Cell Death Dis. 2024 Feb 17;15(2):150. doi: 10.1038/s41419-024-06541-9 (PMC10874437; doi:10.1038/s41419-024-06541-9)
Supplement: Supplementary file 1 — Supplementary Data [file 41419_2024_6541_MOESM1_ESM.pdf]

## SUPPLEMENTARY DATA

**Supplementary Table 1. Identification of candidate proteins whose depletion specifically leads to enhanced radiosensitivity to high-LET protons.**

| Candidate protein | Relative cell survival following high-LET protons | Relative cell survival following low-LET protons |
|---|---|---|
| RAD51 | 0.06 | 1.94 |
| BTG2 | 0.08 | 0.90 |
| PER1 | 0.34 | 1.36 |
| KUB3 | 0.35 | 0.89 |
| MUS81 | 0.40 | 2.42 |
| FLJ10719 | 0.42 | 1.37 |
| BRCA2 | 0.44 | 0.82 |
| POLM | 0.48 | 1.58 |
| REV1L | 0.50 | 1.00 |
| **PARP-1** | **0.54** | **1.06** |
| ERCC5 | 0.55 | 1.10 |
| **OGG1** | **0.55** | **0.82** |
| MGMT | 0.55 | 1.36 |
| TRIM28 | 0.60 | 2.01 |
| SPO11 | 0.67 | 0.84 |
| **PARG** | **0.67** | **1.14** |
| POLB | 0.69 | 0.98 |
| PCNA | 0.69 | 0.84 |

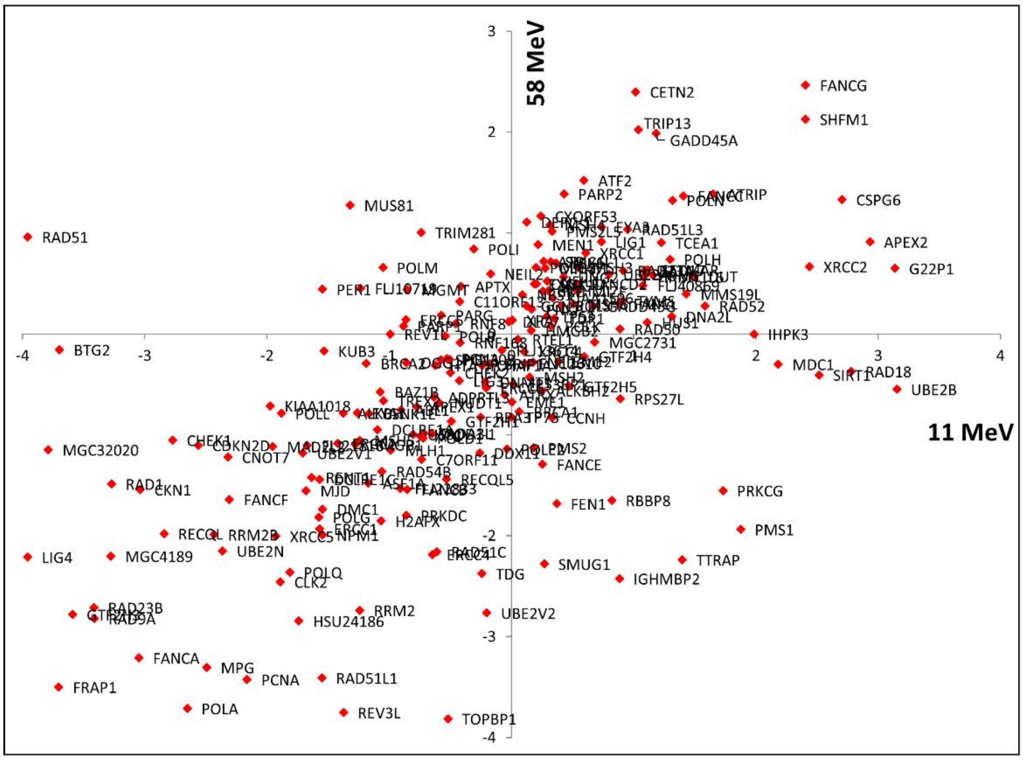


**Supplementary Figure 1. Comparison of cell survival following a DDR siRNA screen in response to high- versus low-LET protons.** HeLa cells were treated with 40 nM of a pool of four siRNAs targeting individual DDR enzymes for 48 h, and irradiated with either 2 Gy high-LET (11 MeV) protons or 2 Gy low-LET (58 MeV) protons. Clonogenic survival of cells was analysed from a single experiment (using triplicate samples) and normalised against the mock treated control which was set to 1.0 (equivalent to ~40 % cell survival post-irradiation). Log2 plots of the data are shown demonstrating fold changes in cell survival in response to high-LET (x-axis) versus low-LET (y-axis) protons.

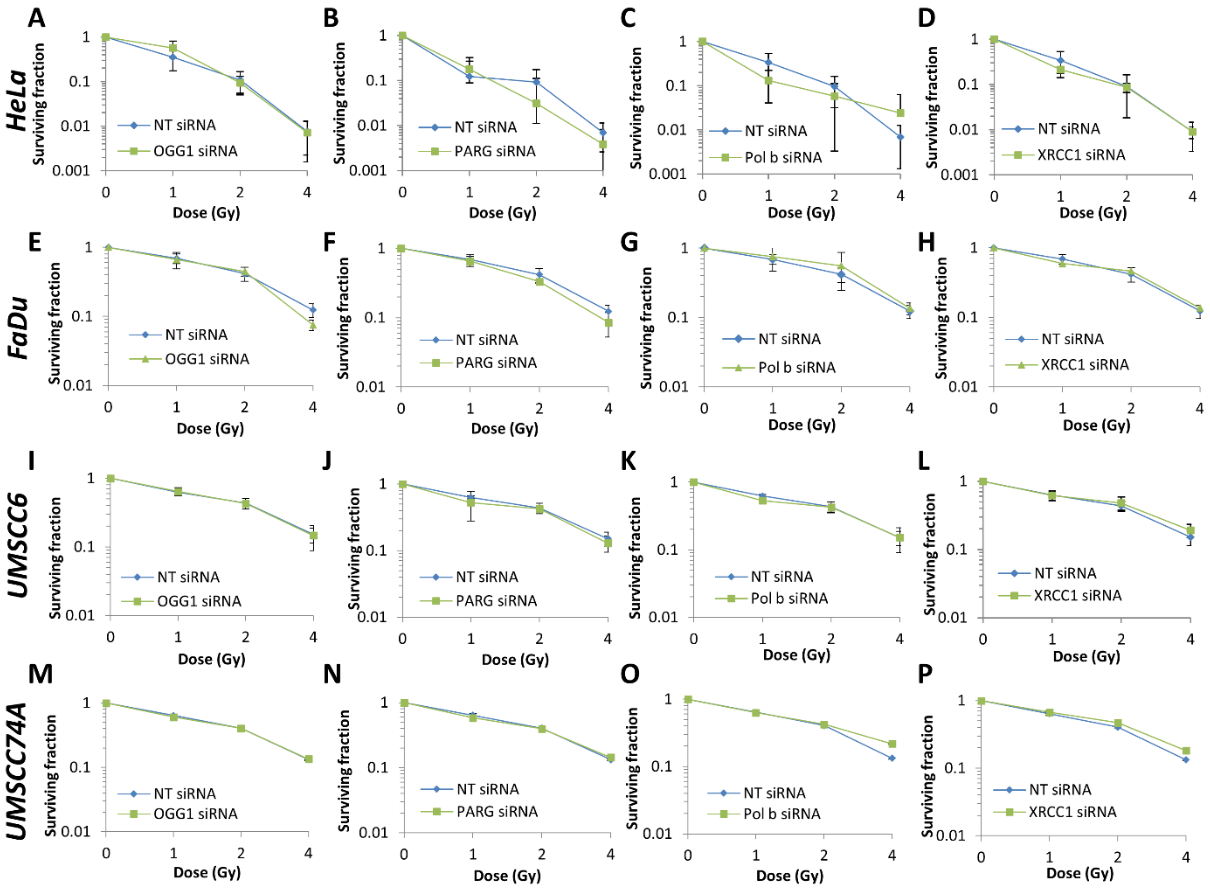


**Supplementary Figure 2. Targeting of OGG1, PARG, Pol β and XRCC1 does not enhance the radiosensitivity of HeLa and head and neck squamous cell carcinoma cells in response to X-ray irradiation.** (**A-D**) HeLa, (**E-H**) FaDu, (**I-L**) UMSCC6 and (**M-P**) UMSCC74A cells were treated with 40 nM of individual siRNAs targeting OGG1, PARG, Pol β, XRCC1 or a non-targeting (NT) control for 48 h. Cells were irradiated with increasing doses of X-rays, and clonogenic survival was analysed from three independent experiments. Shown is the mean surviving fraction with standard errors.

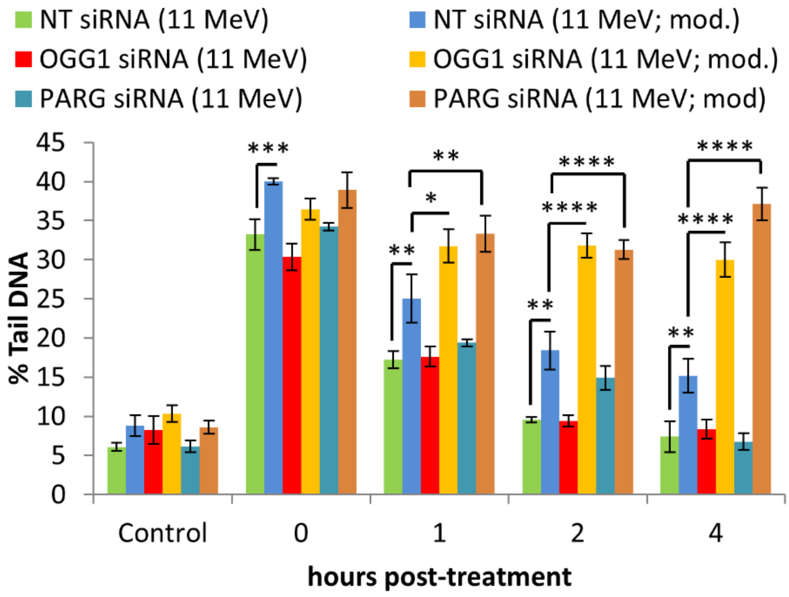


**Supplementary Figure 3. OGG1 and PARG depletion leads to inefficient repair of CDD induced by high-LET protons in head and neck squamous cell carcinoma (UMSCC74A) cells.** UMSCC74A cells were treated with 40 nM of a pool of four siRNAs targeting OGG1 or PARG, versus a non-targeting (NT) control siRNA, for 48 h. Cells were irradiated with 4 Gy high-LET protons and DNA damage measured at various time points post-IR by the enzyme modified neutral comet assay following incubation in the absence (revealing DSBs) or presence (revealing CDD; as indicated by mod) of the recombinant enzymes APE1, NTH1 and OGG1. Shown is the mean % tail DNA±S.D. *p<0.05, **p<0.01, ***p<0.001, ****p<0.0001 as analysed by a one sample *t*-test.

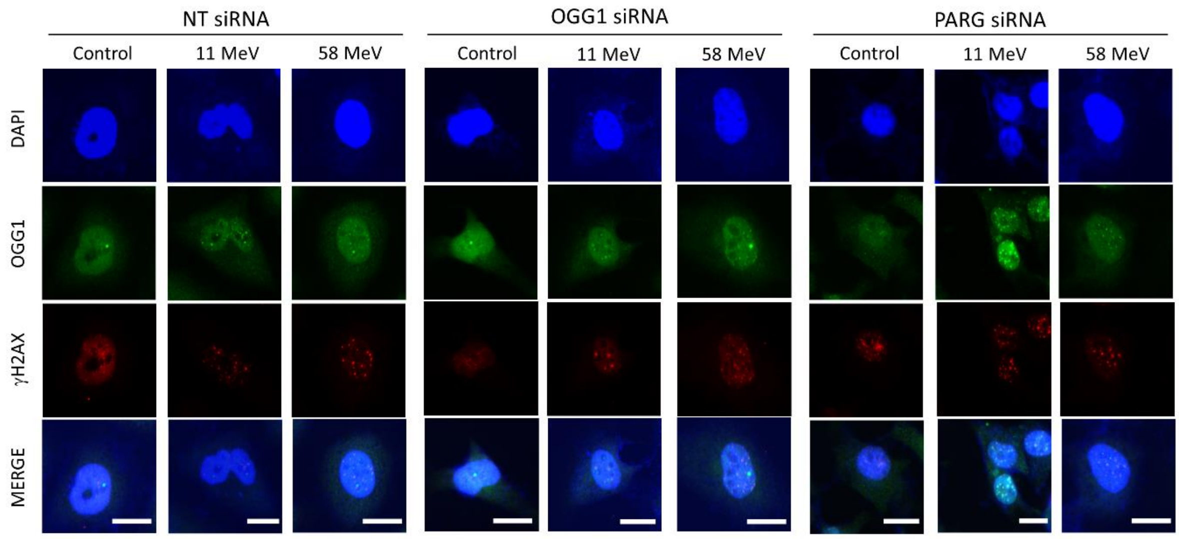


**Supplementary Figure 4. Analysis of γH2AX and OGG1 foci induced by high versus low-LET protons in FaDu cells treated with OGG1 or PARG siRNA.** FaDu cells were treated with 40 nM of a pool of four siRNAs targeting OGG1 or PARG, versus a non-targeting (NT) control siRNA, for 48 h. Cells were either unirradiated (Control), or irradiated with 4 Gy high- or low-LET protons and fixed at 8 h post-irradiation. γH2AX and OGG1 foci were visualised using immunofluorescence microscopy and respective images are shown.

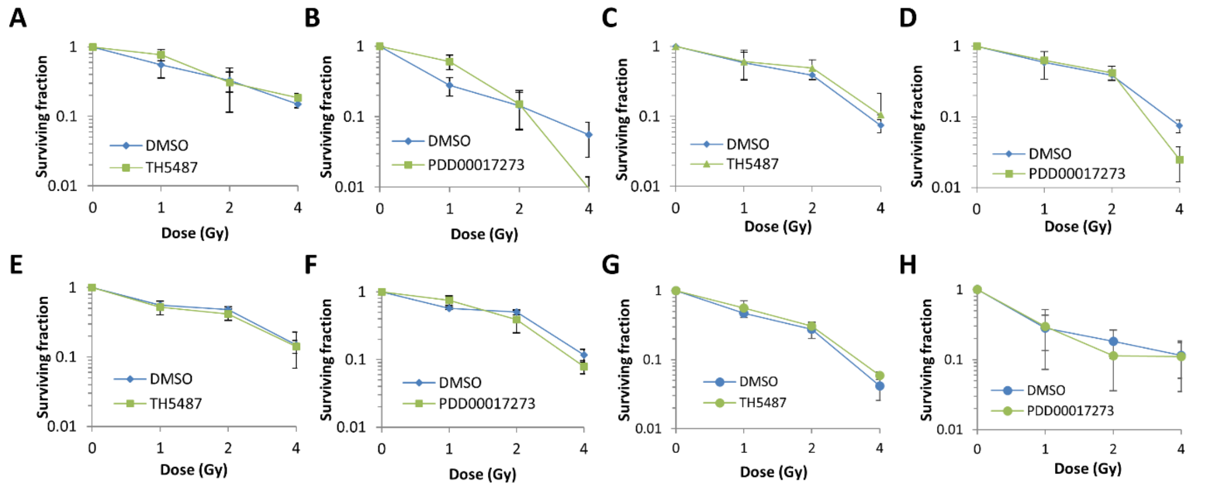


**Supplementary Figure 5. Inhibition of OGG1 or PARG does not enhance the radiosensitivity of HeLa and head and neck squamous cell carcinoma cells in response to X-ray irradiation.** (**A-B**) HeLa, (**C-D**) FaDu, (**I-L**) UMSCC6 and (**M-P**) UMSCC74A cells were treated with 10 µM TH5487, 1 µM PDD00017273 or DMSO for 48 h. Cells were irradiated with increasing doses of X-rays, and clonogenic survival was analysed from three independent experiments. Shown is the mean surviving fraction with standard errors.

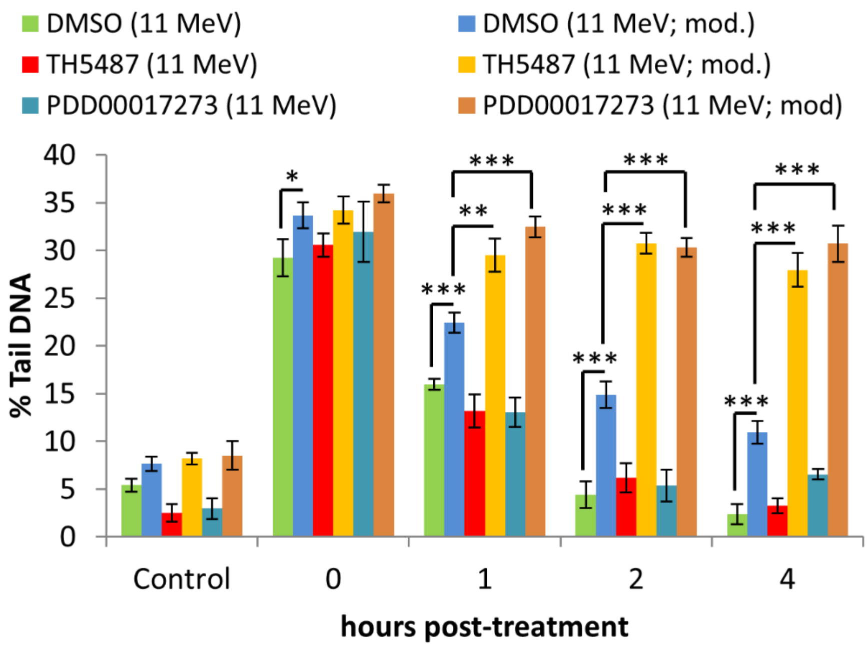


**Supplementary Figure 6. OGG1 and PARG inhibition leads to inefficient repair of CDD induced by high-LET protons in head and neck squamous cell carcinoma (UMSCC74A) cells.** UMSCC74A cells were treated with 10 µM TH5487, 1 µM PDDD00017273 or DMSO for 16 h. Cells were irradiated with 4 Gy high-LET protons and DNA damage measured at various time points post-IR by the enzyme modified neutral comet assay following incubation in the absence (revealing DSBs) or presence (revealing CDD; as indicated by mod) of the recombinant enzymes APE1, NTH1 and OGG1. Shown is the mean % tail DNA±S.D. *p<0.05, **p<0.01, ***p<0.001 as analysed by a one sample *t*-test.

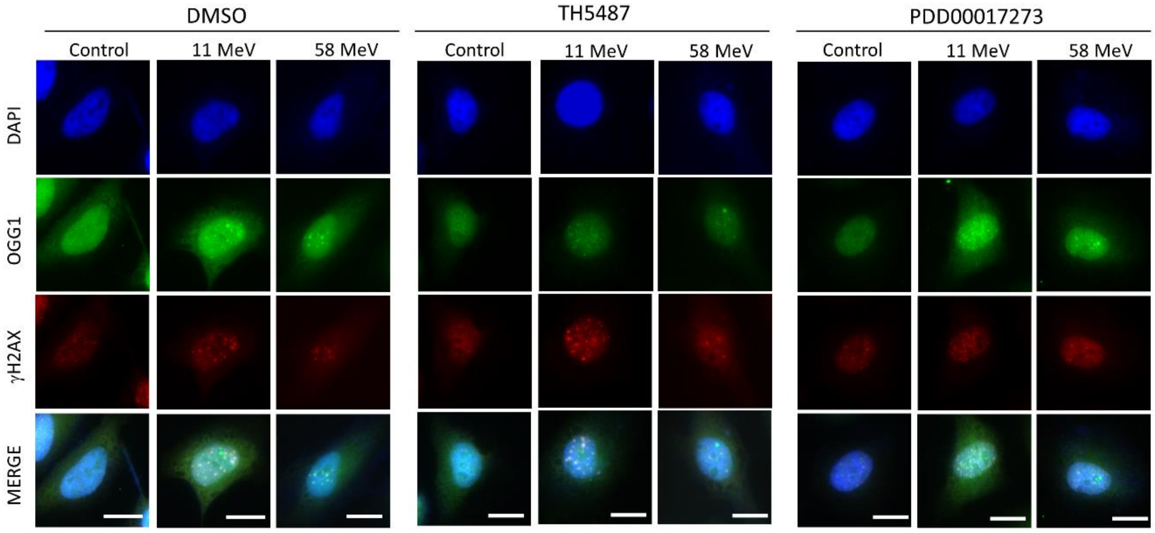


**Supplementary Figure 7. Analysis of γH2AX and OGG1 foci induced by high versus low-LET protons in FaDu cells treated with OGG1 or PARG inhibitors.** FaDu cells were treated were treated with 10 µM TH5487, 1 µM PDDD00017273 or DMSO for 16 h. Cells were either unirradiated (Control), or irradiated with 4 Gy high- or low-LET protons and fixed at 8 h post-irradiation. γH2AX and OGG1 foci were visualised using immunofluorescence microscopy and respective images are shown.

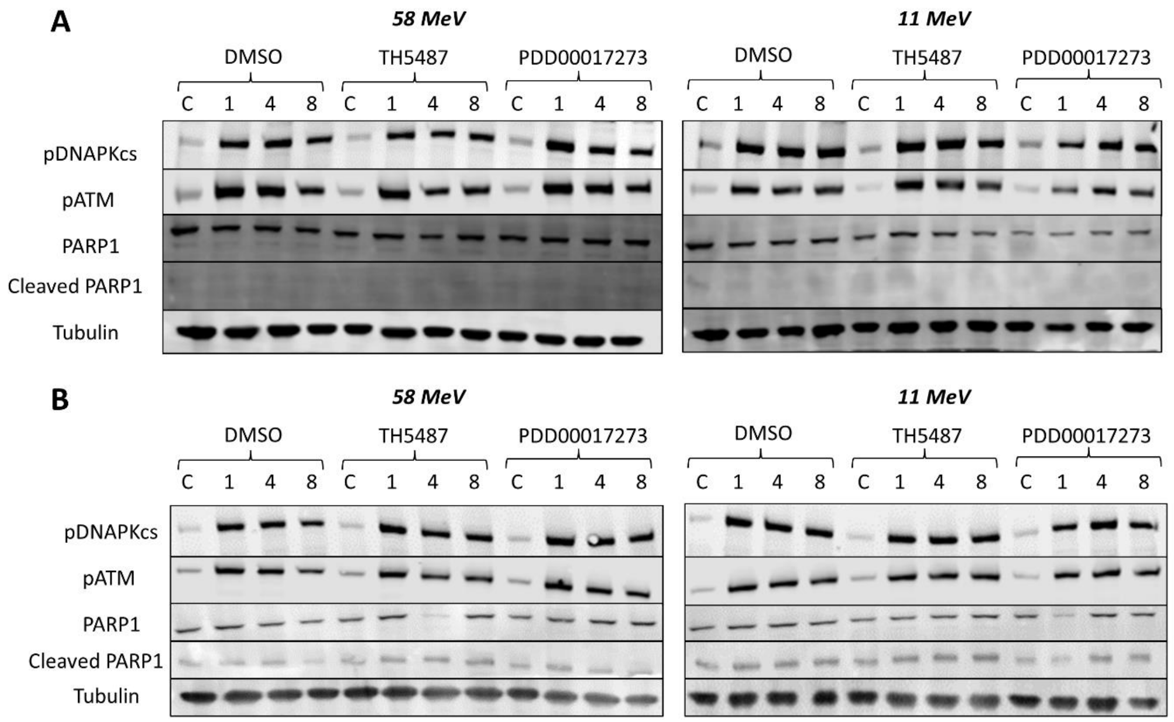


**Supplementary Figure 8. Analysis of key DNA repair proteins following high versus low-LET protons in HeLa and head and neck squamous cell carcinoma cells in the presence of OGG1 and PARG inhibitors.** (**A**) HeLa or (**B**) FaDu cells were treated with 10 µM TH5487, 1 µM PDDD00017273 or DMSO for 16 h. Cells were either unirradiated (Control), or irradiated with 4 Gy high- or low-LET protons and collected at 1-8 h post-irradiation. Whole cell extracts were prepared and analysed by immunoblotting using the indicated antibodies.

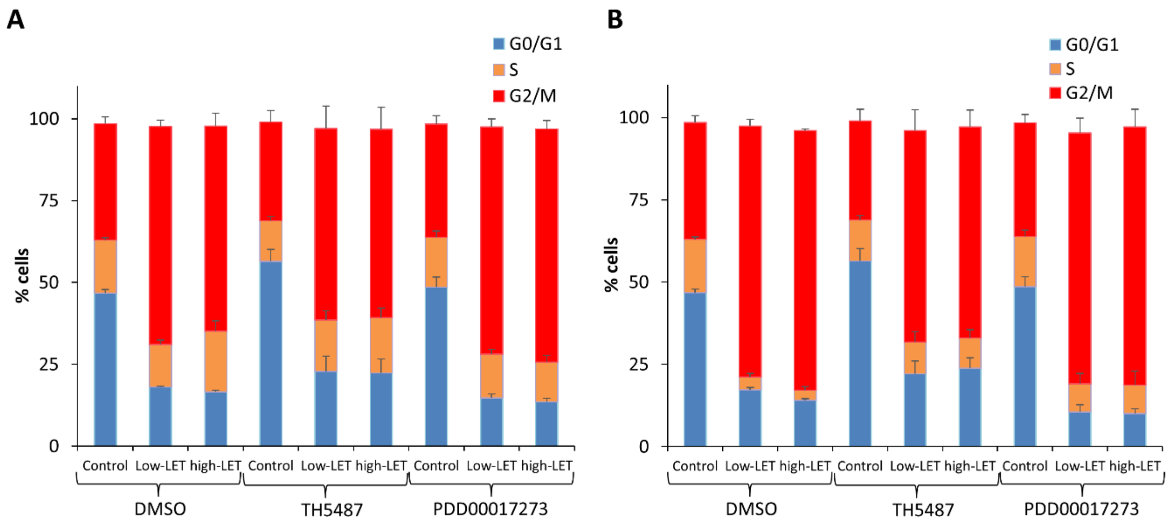


**Supplementary Figure 9. Cell cycle analysis of FaDu cells following high versus low-LET protons in the presence of OGG1 and PARG inhibitors.** FaDu cells were treated with 10 µM TH5487, 1 µM PDDD00017273 or DMSO for 16 h. Cells were either unirradiated (Control), or irradiated with (**A**) 4 Gy or (**B**) 10 Gy high- or low-LET protons, and cells collected at 12 h or 24 h post-irradiation, respectively. Cell cycle analysis was performed using flow cytometry, and shown is the mean % cells with standard errors from three independent experiments.
