# Supplementary material for: Targeting OGG1 and PARG radiosensitises head and neck cancer cells to high-LET protons through complex DNA damage persistence
Source: Cell Death Dis. 2024 Feb 17;15(2):150. doi: 10.1038/s41419-024-06541-9 (PMC10874437; doi:10.1038/s41419-024-06541-9)

Figure 2A

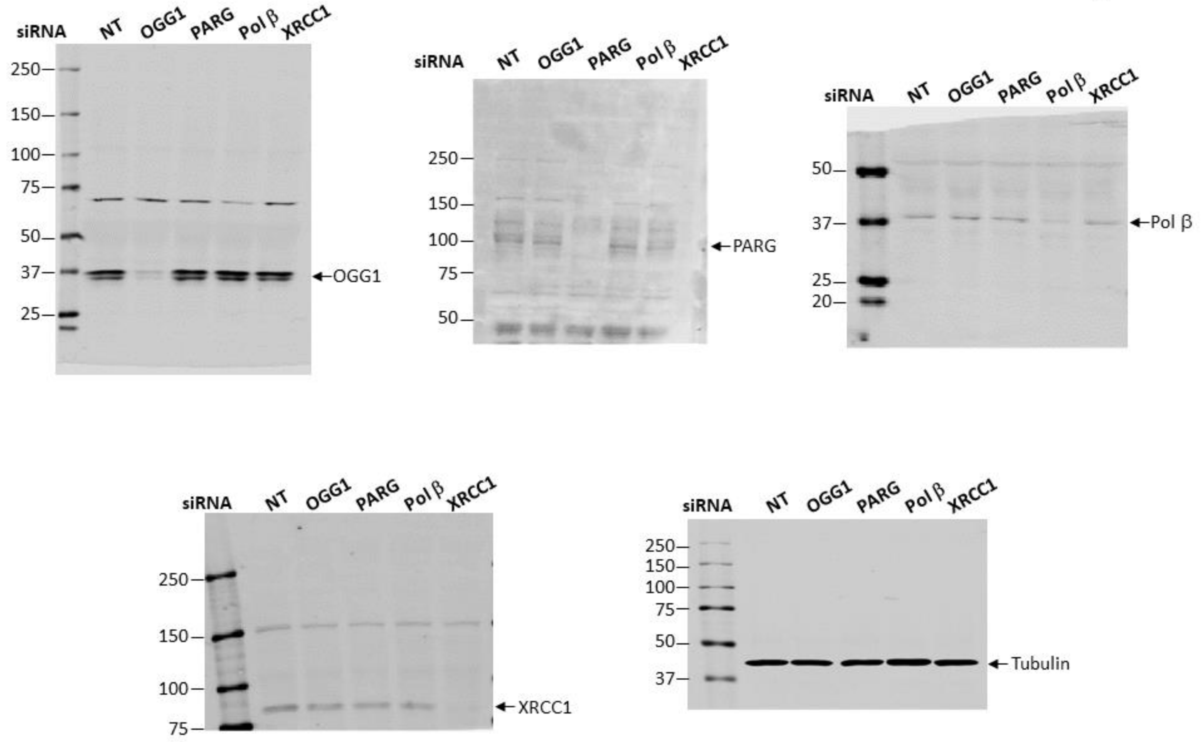
siRNA
NT
OGG1
PARG
Pol β
XRCC1
250
150
100
75
50
37
25
20
OGG1
PARG
Pol β
XRCC1
Tubulin

Figure 3A

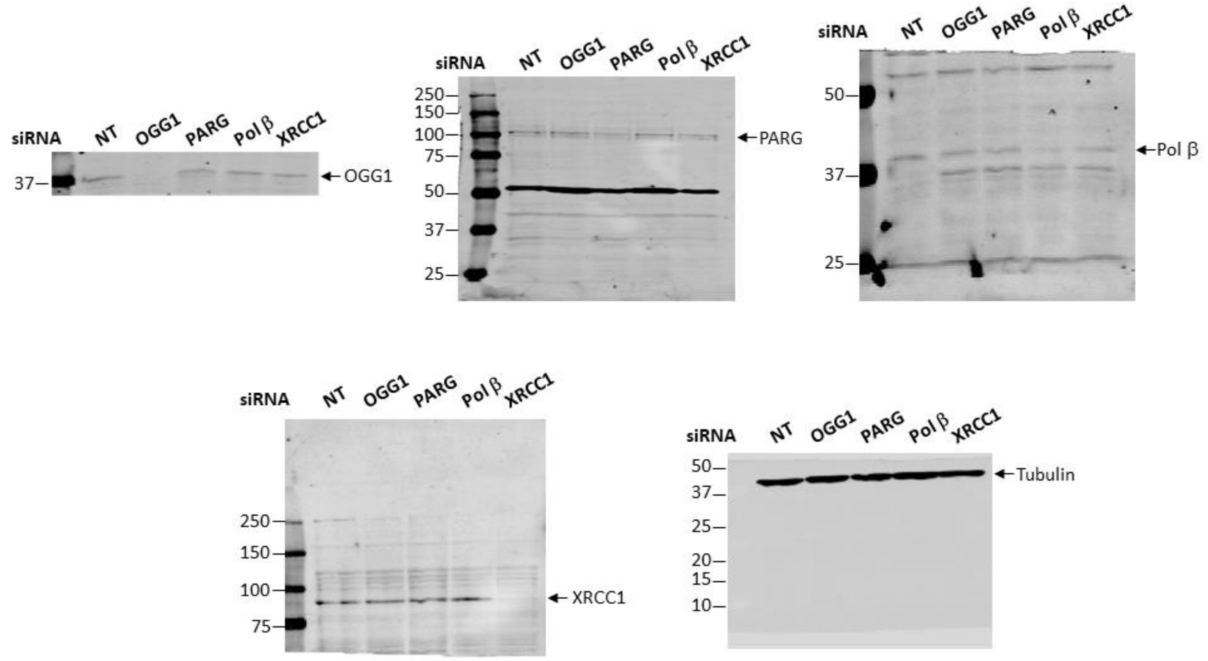
siRNA
NT
OGG1
PARG
Pol β
XRCC1
37
OGG1
siRNA
NT
OGG1
PARG
Pol β
XRCC1
250
150
100
75
50
37
25
PARG
siRNA
NT
OGG1
PARG
Pol β
XRCC1
50
37
25
Pol β
siRNA
NT
OGG1
PARG
Pol β
XRCC1
250
150
100
75
XRCC1
siRNA
NT
OGG1
PARG
Pol β
XRCC1
50
37
25
20
15
10
Tubulin

Figure 4A

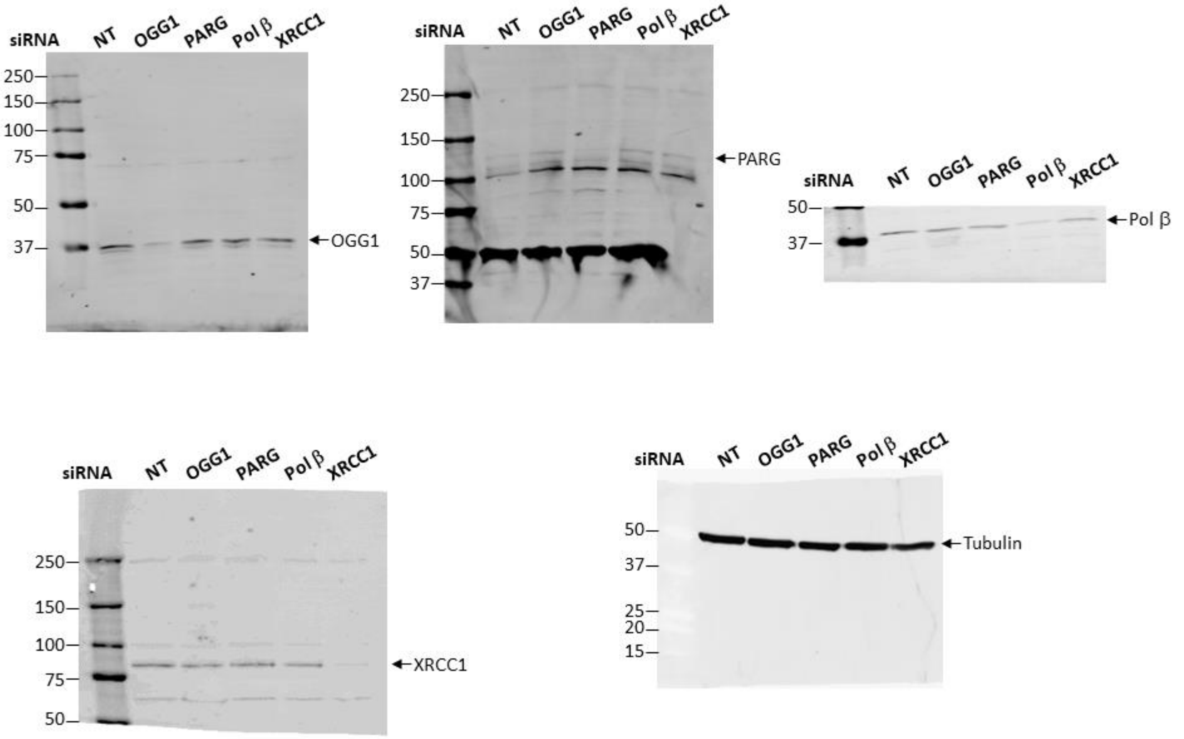
siRNA
NT
OGG1
PARG
Pol β
XRCC1
250
150
100
75
50
37
OGG1
PARG
Pol β
XRCC1
Tubulin
25
20
15

Figure 6H

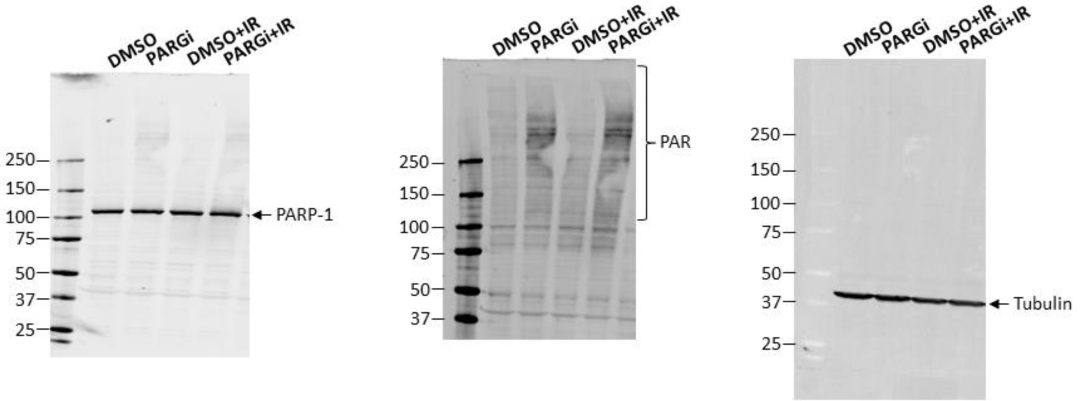
DMSO
PARGi
DMSO+IR
PARGi+IR
250
150
100
75
50
37
25
PARP-1
PAR
Tubulin

Figure 6I

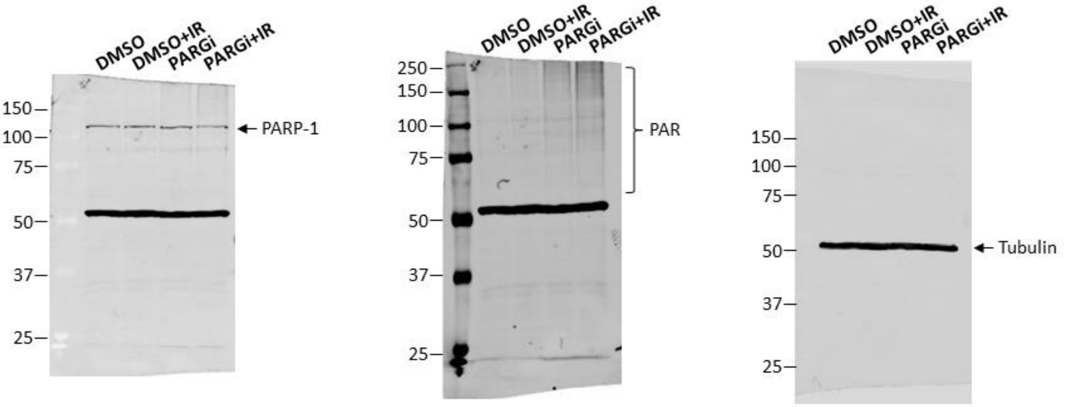
DMSO
DMSO+IR
PARGi
PARGi+IR
150
100
75
50
37
25
PARP-1
250
150
100
75
50
37
25
PAR
DMSO
DMSO+IR
PARGi
PARGi+IR
150
100
75
50
37
25
Tubulin

Figure 6J

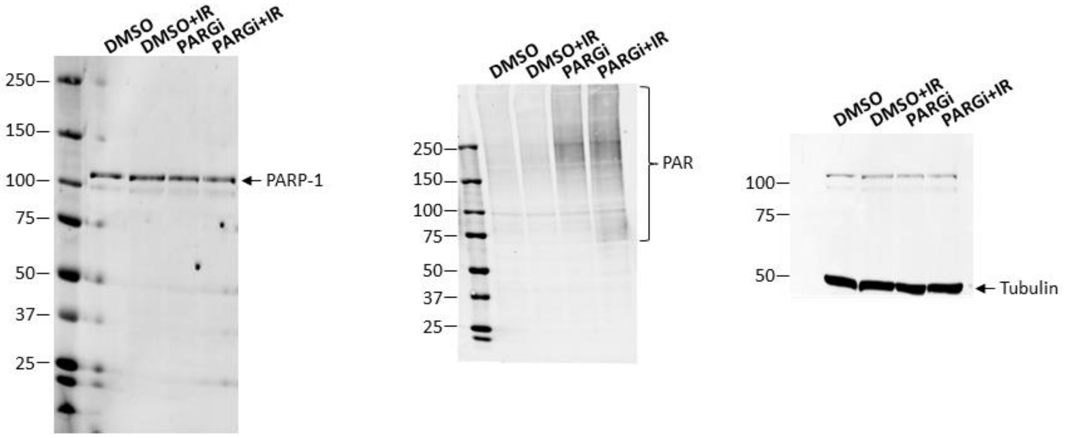
DMSO
DMSO+IR
PARGi
PARGi+IR
250
150
100
75
50
37
25
PARP-1
PAR
Tubulin

Supplementary Figure 8A

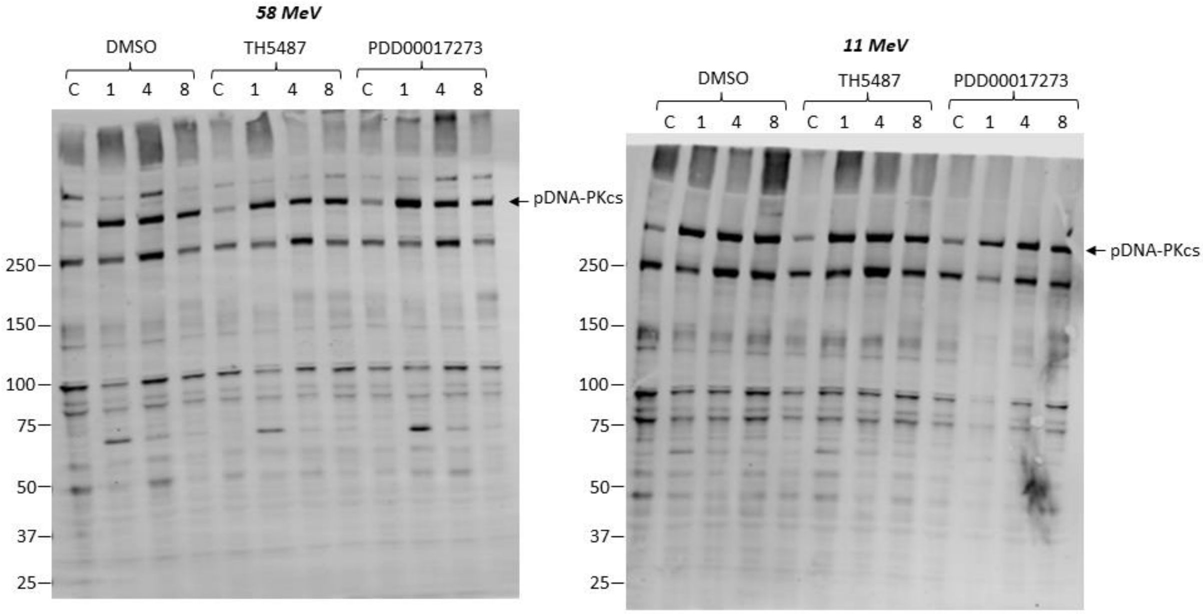

Supplementary Figure 8A

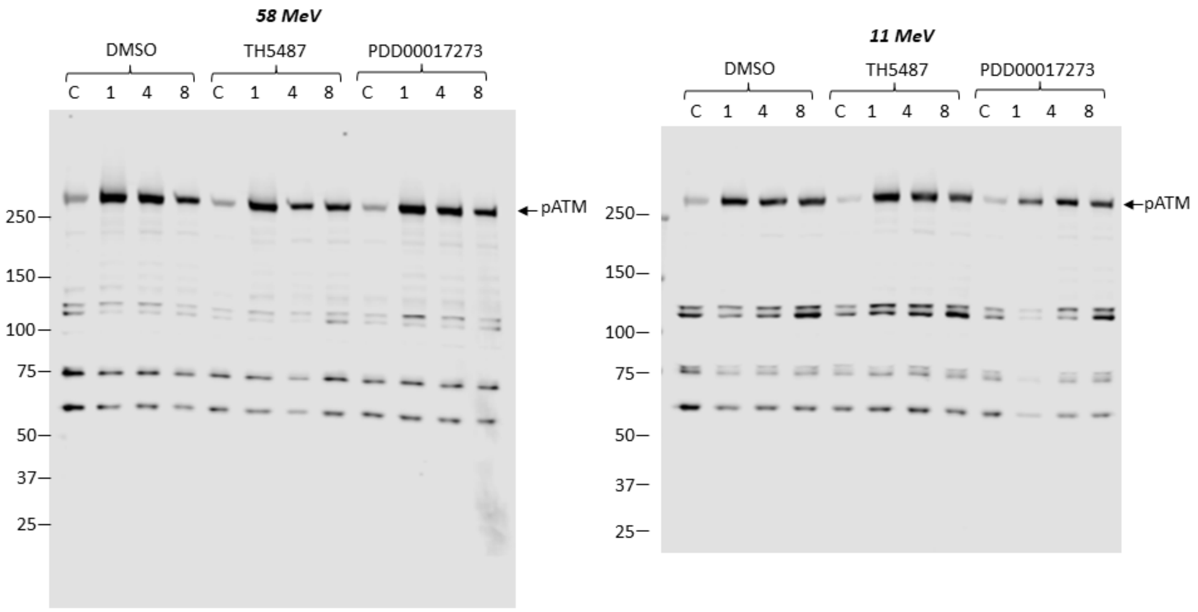

Supplementary Figure 8A

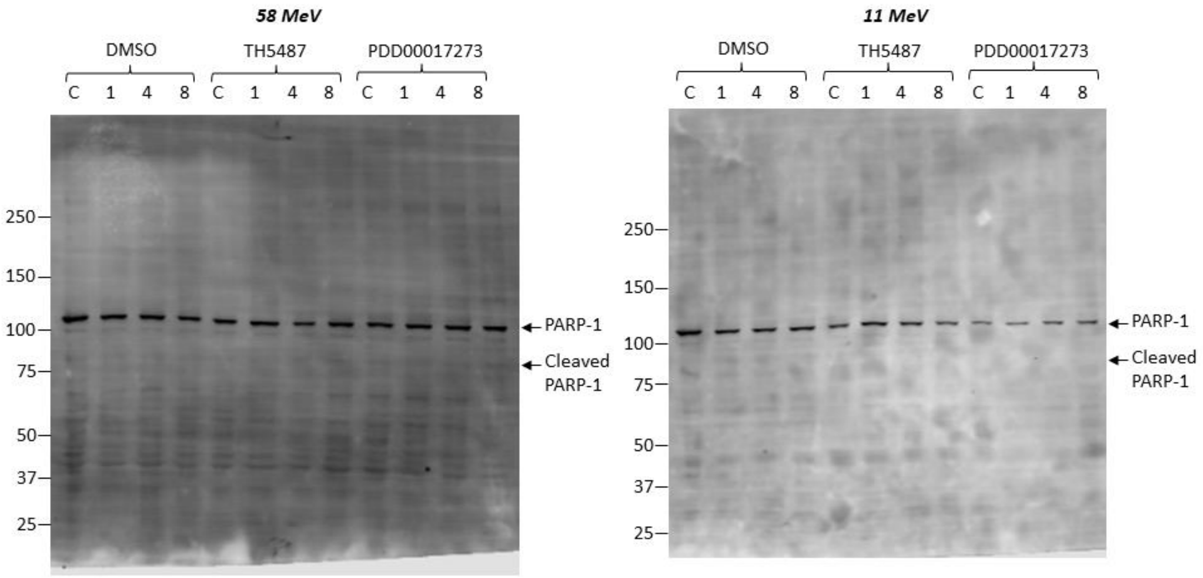

Supplementary Figure 8A

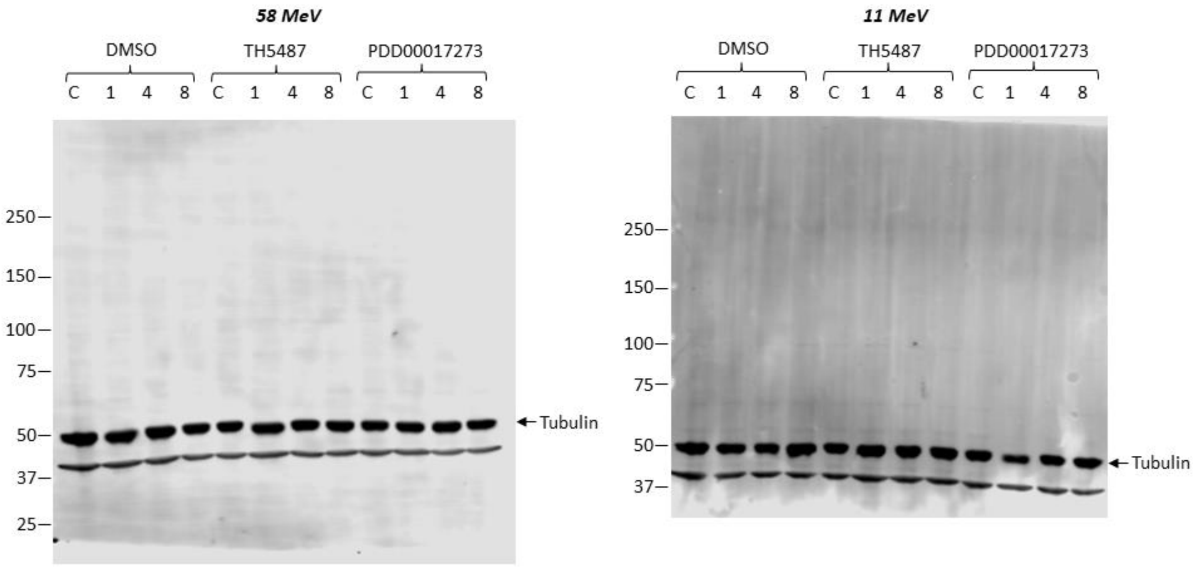

Supplementary Figure 8B

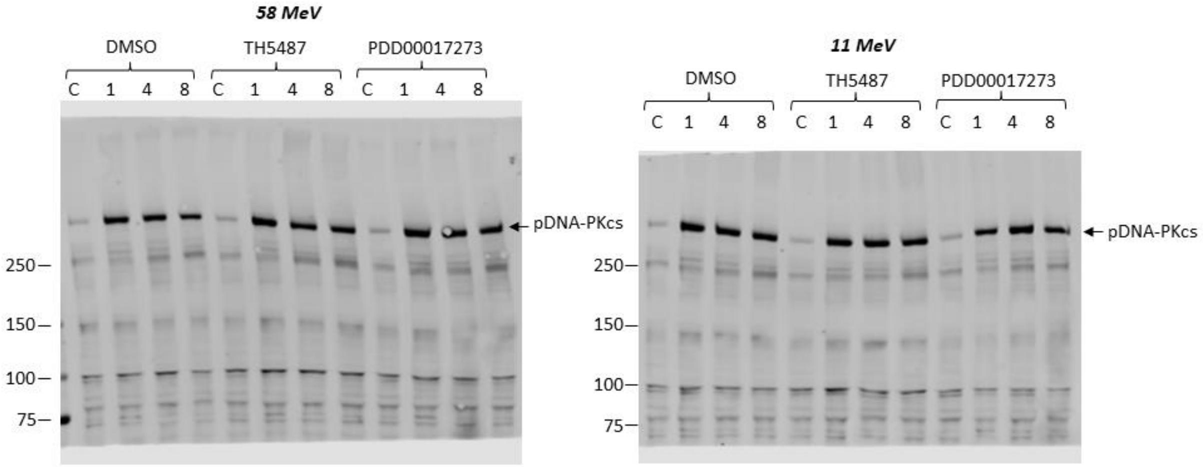

Supplementary Figure 8B

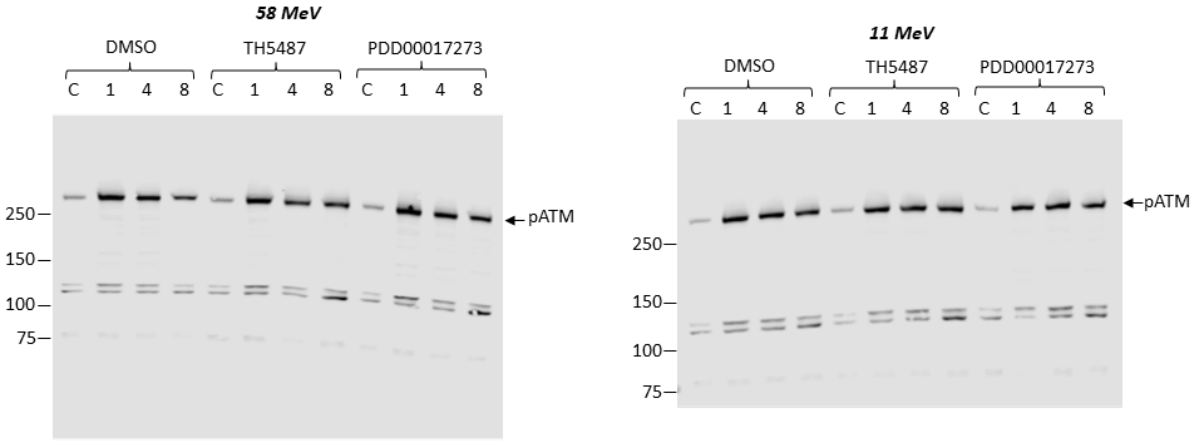

Supplementary Figure 8B

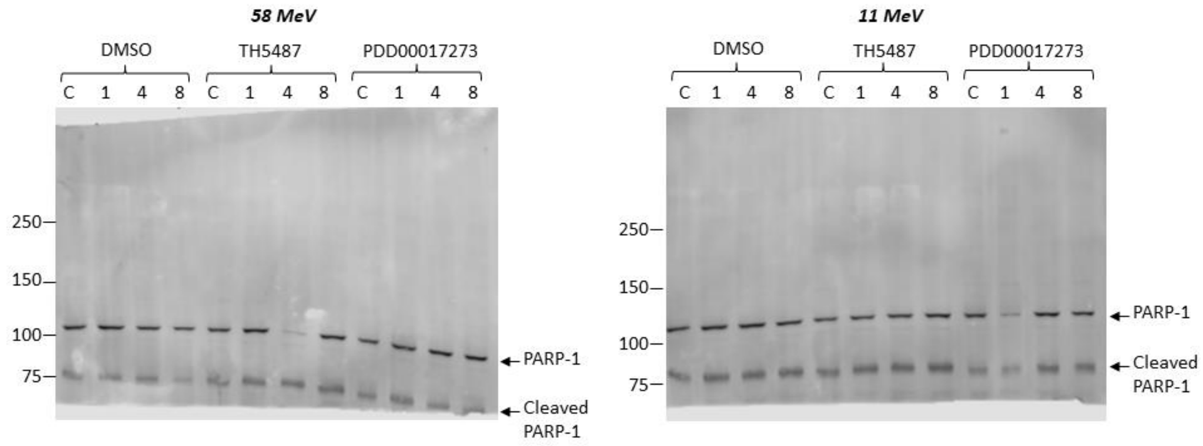

Supplementary Figure 8B

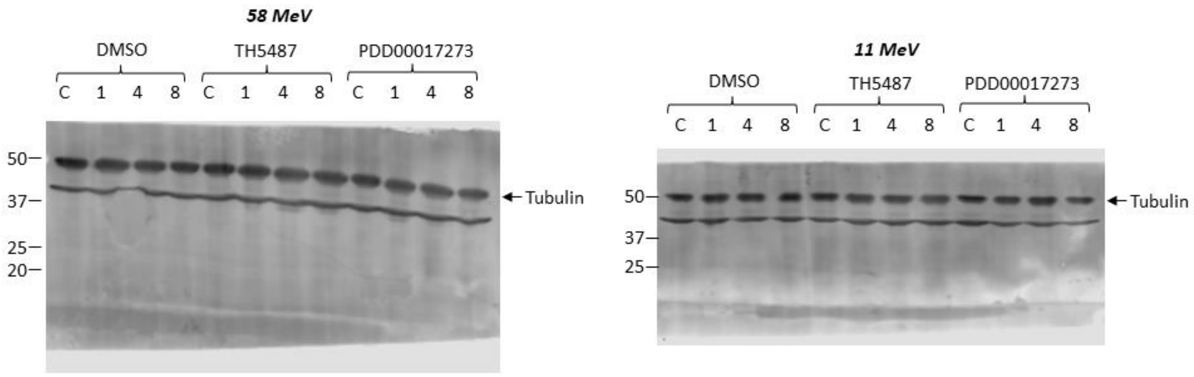

Supplement: Supplementary file 2 — Original Data File [file 41419_2024_6541_MOESM2_ESM.pdf]
